# 3D printed gelatin/decellularized bone composite scaffolds for bone tissue engineering: Fabrication, characterization and cytocompatibility study

**DOI:** 10.1016/j.mtbio.2022.100309

**Published:** 2022-06-06

**Authors:** Aylin Kara, Thomas Distler, Christian Polley, Dominik Schneidereit, Hermann Seitz, Oliver Friedrich, Funda Tihminlioglu, Aldo R. Boccaccini

**Affiliations:** a İzmir Institute of Technology, Department of Bioengineering, İzmir, 35433, Turkey; b Institute of Biomaterials, Department of Material Science and Engineering, Friedrich-Alexander-University Erlangen-Nuremberg, Erlangen, 91058, Germany; c Microfluidics, Department of Mechanical Engineering, University of Rostock, Rostock, 18059, Germany; d Institute of Medical Biotechnology, Department of Chemical and Biological Engineering, Friedrich-Alexander-University Erlangen-Nuremberg, Erlangen, 91052, Germany; e İzmir Institute of Technology, Department of Chemical Engineering, İzmir, 35433, Turkey

**Keywords:** 3D printing, Decellularized bone extracellular matrix, Gelatin, Microbial transglutaminase, Composite scaffolds, Bone tissue engineering

## Abstract

Three-dimensional (3D) printing technology enables the design of personalized scaffolds with tunable pore size and composition. Combining decellularization and 3D printing techniques provides the opportunity to fabricate scaffolds with high potential to mimic native tissue. The aim of this study is to produce novel decellularized bone extracellular matrix (dbECM)-reinforced composite-scaffold that can be used as a biomaterial for bone tissue engineering. Decellularized bone particles (dbPTs, ∼100 ​μm diameter) were obtained from rabbit femur and used as a reinforcement agent by mixing with gelatin (GEL) in different concentrations. 3D scaffolds were fabricated by using an extrusion-based bioprinter and crosslinking with microbial transglutaminase (mTG) enzyme, followed by freeze-drying to obtain porous structures. Fabricated 3D scaffolds were characterized morphologically, mechanically, and chemically. Furthermore, MC3T3-E1 mouse pre-osteoblast cells were seeded on the dbPTs reinforced GEL scaffolds (GEL/dbPTs) and cultured for 21 days to assess cytocompatibility and cell attachment. We demonstrate the 3D-printability of dbPTs-reinforced GEL hydrogels and the achievement of homogenous distribution of the dbPTs in the whole scaffold structure, as well as bioactivity and cytocompatibility of GEL/dbPTs scaffolds. It was shown that Young's modulus and degradation rate of scaffolds were enhanced with increasing dbPTs content. Multiphoton microscopy imaging displayed the interaction of cells with dbPTs, indicating attachment and proliferation of cells around the particles as well as into the GEL-particle hydrogels. Our results demonstrate that GEL/dbPTs hydrogel formulations have potential for bone tissue engineering.

## Graphical abstract

**Figure:**
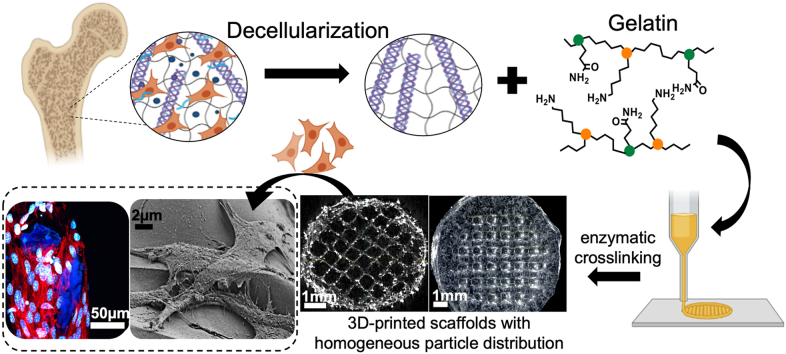

## 1 Introduction

Three-dimensional 3D printing has experienced rapid growth as a newly established field in regenerative medicine [1]. In tissue engineering, 3D printing is a favorable fabrication process due to its ability to control bulk geometry and the internal structure of scaffolds. This technique enables printability of hydrogels as biomaterial ink and thus the formation of a 3D artificial implant or complex tissue ‘‘from the bottom up” in user-defined patterns [2,3]. Hydrogels are widely used as biomaterial inks in 3D printing techniques since their mechanically supportive microenvironment, controllable physical or chemical properties, as well as cellular compatibility allow their use in tissue engineering applications [4,5]. Moreover, hydrogels can mimic the extracellular matrix (ECM) of many tissues with their porous network and provide a suitable microenvironment for cells to migrate and proliferate [6,7]. There are various techniques to fabricate porous hydrogels such as 3D printing [8,9], freeze-drying [[10], [11], [12]], or porogen-based methods [13,14]. Among these methods, 3D printing represents a more robust and controllable method depending on the hydrogel features and printability using layer-by-layer approaches and computer-assisted design to fabricate 3D complex structures [15,16]. In addition, using a combination of these techniques (for instance, 3D printing followed by freeze-drying of the 3D structure) provides the ability to obtain both macro and microporous structures simultaneously. Thus, desirable scaffolds can be produced that better mimic tissues and allow the transfer of nutrients and oxygen to induce cell proliferation and migration.

Additive manufacturing of polymer-based scaffolds has been developed for bone tissue engineering with many advantages. For example, PLA-based 3D-printed scaffolds have been produced with high-resolution capability [17,18]. Incorporation of inorganic fillers into polymer matrices has the potential to produce composite materials with desired physicochemical properties [19]. Distler et al. developed bioactive glass incorporated PLA filaments and demonstrated reproducibility of the produced scaffolds as well as improved mechanical properties and bioactivity [20]. Moreover naturally derived hydrogels exhibit distinct advantages, for instance, controlled degradation, bioactivity, osteogenic capacity as well as the potential for drug delivery applications [[21], [22], [23]]. Among other polymers, gelatin (GEL) is advantageous and preferable in biomaterial designs based on natural polymers due to its nontoxicity, biocompatibility, and biodegradability as well as its arginine-glycine-aspartic acid (RGD) cell recognition sequence in the protein structure [24]. The presence of these sequences improves the biological performance of GEL compared to synthetic polymers that lack RGD cell recognition motifs [25]. Finally, due to its high availability and tunability, GEL is extensively used in medical and pharmaceutical applications and has been recognized as a Generally Regarded As Safe (GRAS) material by the United States Food and Drug Administration (FDA) [26,27]. Moreover, GEL is easily soluble in water and stabilized by various crosslinking strategies [[28], [29], [30]]. It can be easily crosslinked through chemical agents, such as glutaraldehyde [31], genipin [32,33], carbodiimides [34], and enzymatic treatments, for instance with microbial transglutaminase (mTG) [35] and horseradish peroxidases [36]. A better all-around performance in terms of good porosity, compressive strength, cell adhesion, and proliferation was achieved with mTG crosslinking [37]. In 3D printing applications, GEL is a favorable material due to its rheological properties and thermosensitivity [38,39].

Besides the numerous advantages of hydrogels, studies indicated that inferior mechanical properties and low sustainability of hydrogels after printing are the major disadvantages in maintaining the 3D structure for *in vitro* or *in vivo* experiments [40]. To overcome this limitation, mechanical properties can be improved by chemical treatments in the polymer structure, such as block copolymerization [41], formation of inter/semi-penetrating networks [[42], [43], [44]] or addition of fillers, for instance cellulose [45,46], silica nanoparticles [[47], [48], [49]], or using polycaprolactone (PCL) and PLA struts as a supportive structure [50,51]. In addition to using various hydrogels as biomaterial ink, tissues from animals can be treated with different techniques to obtain naturally derived inks for use in regenerative therapy. The design of novel biomaterial inks composed of native ECM components found in bone is an essential approach to developing functional scaffolds that better mimic the biochemistry of the native bone ECM (e.g., collagens, proteoglycans, and enzymes). At this point, decellularization techniques are used to treat tissues by removing their native cellular components without disrupting their histoarchitecture. Thus, decellularized ECM (dECM) can be obtained that can be used in regenerative medicine [[52], [53], [54]]. The main benefit of dECM is preserving the physical features of the tissue retaining components of the natural cell environment to support cell growth during the recellularization process [54,55]. In the present study, bone tissues were decellularized by our new method composed of physical, chemical, and enzymatic treatments. The primary purpose is to preserve both the organic and inorganic components of bone tissue, which is different to currently used decellularization/demineralization methods in bone tissue engineering [56,57]. Following the successful decellularization process, dECM can be processed for various tissue engineering applications [55]. Decellularized bone ECM (dbECM) has been used in different tissue engineering applications including direct use of the dbECM, solubilization of the dbECM as a hydrogel [56], and pulverization to yield particles [58]. Particles can be used as reinforcement of hydrogels, and therefore, can be interesting in new biomaterial ink designs [59]. Combining dbECM as particles composed of both organic and inorganic bone components with biopolymers and hydrogels presents a new approach for bone tissue engineering rather than using dbECM as a standalone scaffold. Thus, dbECM particle reinforced scaffolds with tunable properties can be obtained that provide a natural bone component due to dbECM particles content. Using dbECM as a reinforcement as well as printing dbECM and hydrogel together is the primary motivation of this study. In addition, we assume that an interaction of dbECM particles and GEL via secondary interactions like hydrogen bonding could occur specifically between the inorganic/organic components of dbECM and the GEL residue. To the best of our knowledge, no study has been reported involving dbECM particles reinforced 3D-printed GEL hydrogels that consist of both organic and inorganic components of bone. The advantages of both the dbECM and the biocompatible GEL hydrogel should provide a better scaffold for bone tissue regeneration. We present a minimalistic formulation of a biomaterial ink, which is composed of biocompatible GEL (already available as FDA approved composition) [26,27], and decellularized bone particles (dbPTs), as native sources of collagen and hydroxyapatite, combined with a crosslinking process using naturally derived mTG (already available with FDA approval) [60].

**Fig. 1:**
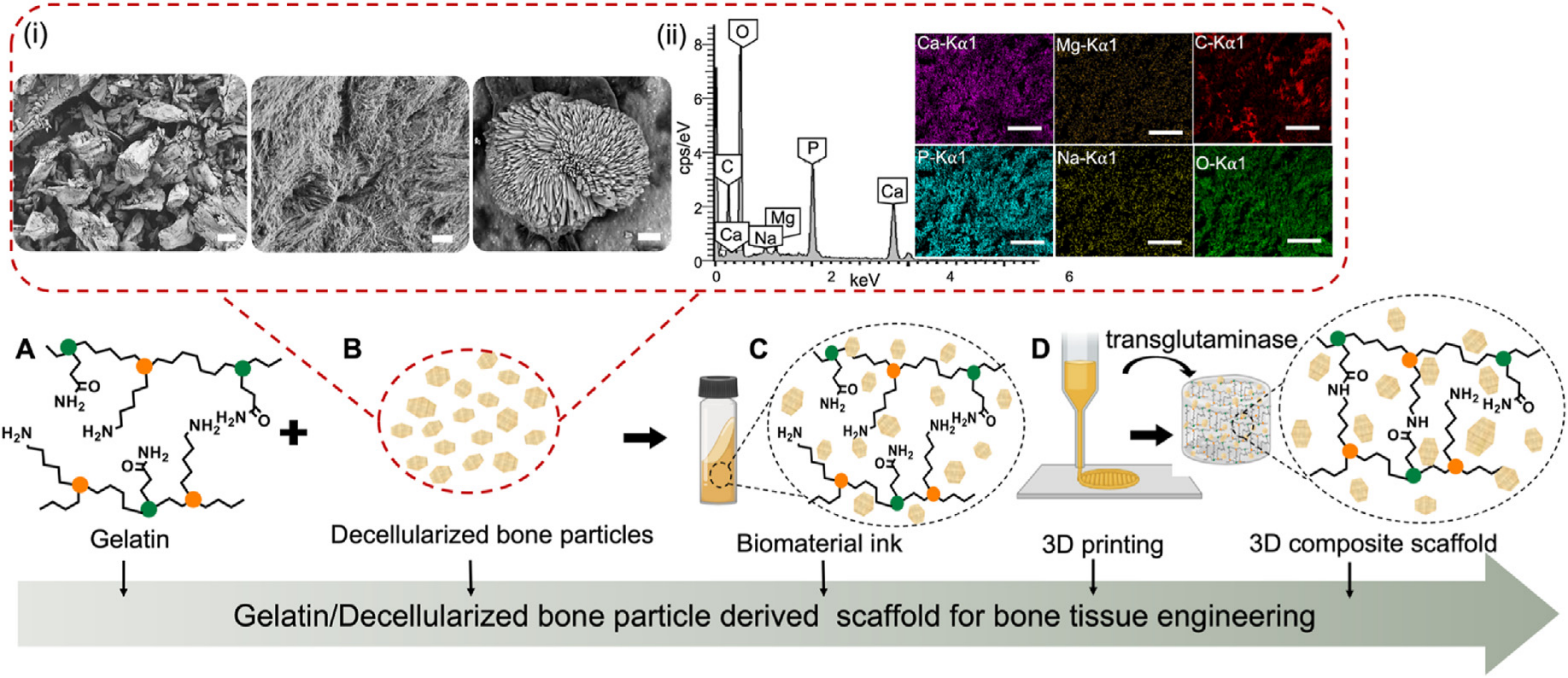
Schematic illustration of GEL-dbPTs composite scaffolds. (A) GEL and (B) dbPTs were used for the preparation of the composite scaffolds. (i) General morphology (left, scale bar: 100 ​μm) of the dbPTs composed of collagen fibers (middle, scale bar: 1 ​μm) and hydroxyapatite crystals (right, scale bar: 1 ​μm) shown in the SEM images. (ii) Energy dispersive x-ray spectrum of the dbPTs indicating the carbon (C), oxygen (O), magnesium (Mg), phosphorus (P), sodium (Na), and calcium (Ca) contents. Scale bars: 500 ​μm. (C) To obtain the biomaterial ink, GEL and dbPTs were mixed, then (D) the GEL/dbPT ink was printed using a 3D extrusion printer. Subsequently, printed scaffolds were cross-linked with mTG by forming the isopeptide bond via lysine and glutamine amino groups. Thus, 3D printed GEL/dbPTs composite scaffolds were produced, and the physicochemical, mechanical properties as well as the cytocompatibility, and cell-material interaction were investigated.

In the present study, we demonstrate the development of porous, 3D-printed composite scaffolds which are composed of GEL and dbPTs (from rabbit) for bone tissue engineering (Fig. 1). dbPTs were mixed with GEL in different concentrations and printed using a 3D-bioprinter to fabricate composite scaffolds. After crosslinking with mTG enzyme, 3D-printed hydrogel scaffolds were freeze-dried to obtain microporous scaffolds, and were characterized morphologically, mechanically, and chemically. Cytocompatibility of the composite porous scaffolds was investigated using MC3T3-E1 mouse pre-osteoblasts for 21 days of cell-culture, to assess the influence of dbPTs on cell growth as well as on the bioactivity of the scaffolds, with potential application in bone tissue engineering.

## 2 Materials and methods

### 2.1 Preparation of the decellularized bone particles

Bone tissue was retrieved from New Zealand White Rabbit's femur (weight, 2.5–3.0 ​kg; female) in accordance with the guidelines approved by the Animal Experiments Ethical Committee of the Dokuz Eylul University School of Medicine, İzmir, Turkey (Protocol No:44/2019). Bone tissue was decellularized by the combination of physical, chemical, and enzymatic methods. First, tissue was treated in freeze-thaw cycles then incubated in a hypotonic buffer at 37 ​°C for 24 ​h. 0.1% (w/v) sodium dodecyl sulphate (SDS) in the presence of 0.1% (w/v) ethylenediaminetetraacetic acid (EDTA) was used for removing the cellular components from the tissue at 45 ​°C for 48 ​h. For the enzymatic treatment, samples were incubated twice in a nuclease solution consisting of DNase I in 50 ​mM Tris-HCl and 50 ​mg/ml BSA buffer for 4 ​h at 37 ​°C. Hypertonic buffer was used as a final incubation step. Then, samples were washed with 1X PBS for 24 ​h at room temperature. For sterilization, samples were incubated with 0.01% peracetic acid for 3 ​h. Finally, bone tissue was washed in PBS at 37 ​°C and 25 ​°C for 24 ​h, respectively.

After the decellularization process, tissue samples were pulverized using a laboratory mixer and filtered with 100 ​μm mesh diameter as dbECM microparticles.

### 2.2 Verification of the decellularization process

The decellularization process was verified in terms of histological, morphological, and biochemical aspects. In all evaluations adjacent pieces of the bone samples were used as control.

For the histologic evaluation, tissues were fixed with 4% (v/v) paraformaldehyde for 72 ​h then dehydrated in ethanol, acetone, and xylene and embedded in paraffin. 5 ​μm thick sections were prepared by using a microtome (Histocore Multicut, Leica Biosystems) and hematoxylin eosin (HE) staining was used to evaluate general histoarchitecture of bone tissue.

For SEM imaging, bone samples were fixed with paraformaldehyde for 20 ​min at room temperature. Following a washing process with 1xPBS, samples were dehydrated in graded ethanol series (50%, 70%, 80%, 90% and 100%). The samples were then observed by SEM (Quanta FEG, Thermo Fisher Scientific).

To quantify the total DNA content, bone samples were homogenized using DNeasy 96 Blood & Tissue Kit (Macherey-Nagel, GmbH&Co KG), following the manufacture's protocol, then DNA content of tissues was measured at 260/280 ​nm in a Nanodrop spectrophotometer (Nano 2000, Thermo Scientific). The total collagen content of the samples was measured based on hydroxyproline assay according to the Quickzym (Bioscience) kit manual. The absorbance at 570 ​nm was recorded by a microplate reader (Varioskan Flash, Thermo Fisher Scientific). The concentration of hydroxyproline was estimated by interpolation from a hydroxyproline standard curve.

### 2.3 Preparation of the gelatin-decellularized bone particles precursor

GEL solution was prepared by dissolving 15% (w/v) GEL (from porcine skin, Type A, Bloom 300, Sigma) in ultrapure water at 80 ​°C for 3 ​h and stored at +4 ​°C until further use. dbPTs were prepared by dispersing in ultrapure water under stirring at 37 ​°C. Equal volumes of GEL and dispersed particles were mixed at room temperature for 15 ​min, resulting in a final concentration of 7.5% GEL and 1%, 3% and 5% (w/v) dbPTs by stirring. To reach the optimum viscosity, the cylindrical rotator (Intelli-Mixer, ELMI, Latvia) was used until the dbPTs became stable in the GEL matrix prior to 3D printing.

### 2.4 3D printing

3D cylindrical scaffolds were fabricated using a 3D extrusion printer Gesim Bioscaffolder (GeSiM, GmbH, Germany). In order to stabilize the viscosity, the cartridge holder temperature was set to 25 ​°C. The GEL/dbPTs hydrogel precursor was transferred into the cartridge, inserted into the holder, then extruded through the 400 ​μm nozzles with a tip velocity of 5 ​mm/s and extrusion pressure of 120–180 ​kPa. During 3D printing, extrusion pressure was set according to the filament formation. All samples were fabricated from 10 layers with one layer height set to 0.3 ​mm, and the diameter was 10 ​mm. The printed scaffolds were crosslinked using 10% w/v microbial mTG from *Streptoverticillium mobaraense* (Ajinomoto Co., Inc., ACTIVA WM, 85–135 U/g); initially 15 ​min at room temperature, then further overnight at +4 ​°C, then lyophilized by freeze-drying (LD1-2 Plus, Martin Christ GmbH, Germany) for 48 ​h.

### 2.5 Printability assessment

To determine the accuracy of printing, light microscopy (Stemi 508, Carl Zeiss, Germany) was used to obtain images of the 3D printed GEL/dbPTs scaffolds. The images were processed using Image J software. Printability factor (Pr) as function of the pore circularity (C), pore perimeter (P) and pore area (A) was calculated using the following equation [61]:

(1) $$\text{Pr}=\frac{\pi }{4}\text{x}\frac{1}{\text{C}}=\frac{{\text{P}}^{2}}{16\text{A}}$$

The uniformity of the printed struts was determined using the previously described uniformity factor U [62], which equals to the measured horizontal length of a printed hydrogel strut (L) divided by the theoretical horizontal length of a parallel printed strut (L_t_) as shown in Eqn (2);

(2) $$\text{U}=\frac{\text{L}}{{\text{L}}_{\text{t}}}$$

### 2.6 Rheological characterization

The rheological properties of the dbPT-reinforced inks were determined using a rotational rheometer MCR 702 equipped with a plate-plate geometry with a diameter of 25 ​mm (Anton Paar, Graz, Austria). The linear viscoelastic range and the yield point were carried out by an amplitude sweep in a deformation range of 0.0001–10 ​at a frequency of 10 ​rad/s and a temperature of 25 ​°C. The thermo-responsiveness of the precursors was determined by a temperature sweep from 37 to 20 ​°C at a frequency of 10 ​rad/s. To describe the structural recovery, a thixotropy test was applied. The test is divided into a 30 ​s transient phase, a 30 ​s loading phase, and a 120 ​s recovery phase. During the transient and recovery phases, the precursor was subjected to deformation of 0.001 ​at 10 ​rad/s. For the loading phase, to destroy the structure, the deformation was increased to 6 ​at a frequency of 10 ​rad/s. The entire thixotropy test was carried out at 25 ​°C. To prevent drying of samples, and to ensure a homogeneous heat distribution, all tests were performed with a Peltier plate at a gap size of 0.5 ​mm.

### 2.7 Scanning electron microscopy (SEM)

The surface morphology, pore structure and particle-hydrogel interaction of the 3D-printed scaffolds were assessed using SEM. Freeze-dried samples were coated with a thin gold layer prior to analysis, and SEM images were recorded with a scanning electron microscope (Auriga CrossBeam, Carl Zeiss, GmbH, Germany).

### 2.8 X-ray microtomography (μCT)

To investigate the dbPT distribution in 3D-printed GEL-dbPTs scaffolds, μCT analysis was performed. The tomograms of the scaffolds were recorded on a Skyscan 1076 scanner (Bruker, Kontich, Belgium), applying a source voltage of 37 ​kV and a source current of 228 ​mA. To reduce beam hardening artifacts, a 0.025 ​mm titanium filter was used. The scan resolution was set to 9 ​μm per voxel. For noise reduction, an average of 4 frames was recorded every 0.3°. The scans were reconstructed applying the cone-beam algorithm in the NRecon software package (Bruker, Kontich, Belgium). The datasets were segmented globally, and the lower gray threshold was set to 155 and the upper gray threshold to 255 to account for the denser dbPTs. A 3D analysis of the segmented datasets was performed to determine the particle number and particle size distribution. For the segmentation and 3D analysis, the software CT analyser (Bruker, Kontich, Belgium) was used. High-resolution 3D renderings were created using CTVox software (Bruker, Kontich, Belgium).

### 2.9 Fourier transform infrared spectroscopy (FTIR)

The chemical composition of the GEL/dbPTs scaffolds was evaluated by Attenuated Total Reflectance FTIR (ATR-FTIR) analysis at a wavenumber range of 4000 to 400 ​cm^−1^ (IRAffinity-1S, Shimadzu, Japan).

### 2.10 Swelling/degradation kinetics of GEL/dbPTs scaffolds

The swelling/degradation properties of the 3D-printed scaffolds were investigated by weight changes during 56 days of incubation period. Samples were incubated in degradation medium which was similar to the maintenance medium in cell culture composed of alpha-modified minimum essential medium (α-MEM, Gibco, Life Technologies™, Germany), supplemented with 1% (v/v) l-glutamine, 10% (v/v) Fetal Bovine Serum (FBS) and 1% (v/v) penicillin-streptomycin (all supplements: Sigma Aldrich, Germany) at 37 ​°C with 5% CO_2_ and 95% relative humidity, and the medium was refreshed every 48 ​h. The samples (n ​= ​6 per group) were immersed into the medium and weighed after careful removal of the excess medium at each specific time point. The initial mass of the samples prior to immersion in medium (m_i_) and the current weight at each time point (m_c_) were recorded. Swelling and degradation were calculated in weight % by the following equation:

(3) $$swelling\;\left(wt\%\right)\;or\;degradation\;\left(wt\%\right)=\left(\frac{m_{c}-\;m_{i}}{m_{i}}\right)x100$$

### 2.11 Mechanical characterization

The mechanical properties of the GEL/dbPTs scaffolds were evaluated by using uniaxial unconfined compression tests using an Instron 5967 universal testing machine equipped with a 100 ​N load cell (Instron® GmbH, Germany) in accordance to previously published protocols [63,64]. The tests were carried out using six replicates (n ​= ​6) of cylindrical hydrogel samples (diameter ​= ​3 ​mm, height ​= ​7 ​mm). Compression loading was performed at 1 ​mm/min deformation speed up to 15% strain. The Young's modulus of the scaffolds was determined as the slope in the linear-elastic deformation region of stress-strain diagrams between 5% and 10% strain. Stress-relaxation time of the samples was defined as the time after which 75% of the initial stress dissipated in the samples, similarly to as described before [63,65].

### 2.12 *In vitro* cell culture study

#### 2.12.1 Cell culture

Pre-osteoblast MC3T3-E1 cells (Sigma Aldrich, Germany) were used to assess the cytocompatibility of 3D-printed GEL/dbPTs scaffolds. The cells (passage 5, P5) were sub-cultured in α-MEM supplemented with 1% (v/v) l-glutamine, 10% (v/v) FBS and 1% (v/v) penicillin-streptomycin in an incubator at humidified atmosphere of 5% CO_2_, 95% humidity and 37 ​°C. 3D-printed scaffolds were disinfected under UV light exposure for 1 ​h (for each side of the scaffolds), then immersed in the culture medium for conditioning. Cells at P8 (1 ​× ​10^5^ ​cells/scaffolds) were seeded on 3D-printed scaffolds and cultured at 37 ​°C in a humidified atmosphere of 95% air and 5% CO_2_ in an incubator. Cylindrical 10 layered GEL/dbPTs composite scaffolds containing 1% and 3% (wt) dbPTs were used for all cell culture studies. The pure GEL scaffolds served as material control, and tissue culture polystyrene (TCP) seeded cells served as an additional control.

#### 2.12.2 Cell viability

Water-soluble tetrazolium salt (WST-8) assay was performed to determine the viability of cells on the scaffolds by conversion of water-soluble tetrazolium salt through cellular metabolism into insoluble formazan. MC3T3-E1 cell-seeded GEL/dbPTs scaffolds (n ​= ​4) were cultured for 21 days and at each time point, the medium was removed, and cell-seeded scaffolds were incubated with WST-8 solution (Cell Counting Kit-8, Sigma Aldrich, Germany) for 3 ​h according to the manufacturer's instructions. After incubation, 100 ​μl aliquots were transferred into a 96-well-plate, and the absorbance at 450 ​nm was recorded using a plate reader (PHOmo, Anthos Mikrosysteme GmbH, Friesoythe, Germany).

#### 2.12.3 Live/dead staining

The cellular viability in the 3D-printed scaffolds was determined by a Live/Dead staining assay. Initially, cell-seeded scaffolds were washed with Hank's Balanced Salt Solutions (HBSS) and incubated in HBSS containing 4 ​μl/ml Calcein AM and 5 ​μl/ml PI (Invitrogen, Molecular probes by Life Technologies, USA) for 45min at 37 ​°C, 5% CO_2_ in a humidified atmosphere. To stain the cell nuclei, 1 ​μl/ml DAPI (4′,6-diamidino-2-phenylindole, Invitrogen, USA) was used. After incubation, samples were washed with HBSS and examined by fluorescence microscopy (AxioScope A.1, Carl Zeiss, Germany), visualizing cell nuclei (blue), live (green) and dead (red) cells in the scaffolds.

#### 2.12.4 Extracellular lactate dehydrogenase release assay (LDH)

The potential cytotoxicity of the scaffolds was determined using the LDH kit (Tox7 Toxicity kit, Sigma Aldrich). Cell culture medium was removed from samples and mixed with substrate solution, LDH cofactor solution and dye solution into the cuvettes. Following the incubation of the samples at RT for 30 ​min in the dark, the absorbance at 490 and 690 ​nm was measured using a UV–vis spectrophotometer.

#### 2.12.5 PicoGreen assay

The proliferation of the cells on the scaffolds was determined based on quantifying the double-strand DNA (dsDNA) by Quant-iT PicoGreen ds-DNA Assay-Kit (Invitrogen, Life Technologies, Thermo Fisher, USA). Cell-seeded 3D-printed GEL/dbPTs scaffolds (n ​= ​4) were incubated for 28 days of culture period. The samples were washed with PicoGreen assay buffer solution, then mixed with a working solution provided by the kit and incubated for 5 ​min at room temperature, protected from light. The relative fluorescence (RFU) was recorded using a CFX connect spectrofluorometer (Bio-Rad, Germany).

#### 2.12.6 Multiphoton microscopy

In order to assess the cell orientation, cell-seeded scaffolds were examined by multiphoton microscopy. Samples (n ​= ​3) were fixed using 4% formaldehyde for 5 ​min in the dark and washed with HBSS. 0.1% Triton X-100 was used for permeabilization. Then samples were washed twice using HBSS. Samples were stained with, first, 5 ​μl/ml F-actin (Rhodamine Phalloidin F-Actin, Thermo Fisher Scientific, USA) for 1 ​h and, second, 1 ​μl/ml DAPI (Thermo Fisher Scientific, USA) for 5 ​min. The samples were examined using a multiphoton microscope (TriMScope II, LaVision BioTec, Bielefeld, Germany), equipped with an HC FLUOTAR L 25x/0.95 ​W VISIR objective. The images were recorded at 810 ​nm excitation, acquiring DAPI at 450/70 ​nm (ET450/70 ​m) and Phalloidin at 620/60 ​nm (ET620/60 ​m). Multiphoton microscopy data were processed via ImageJ (v1.53f51), and 3D renders were created using the 3Dscript plugin [66].

#### 2.12.7 SEM analysis

Morphology, distribution and spreading of the cells on the 3D-printed GEL/dbPTs scaffolds were investigated by SEM analysis. Initially, cells were fixed using a fixation solution containing 3% (v/v) glutaraldehyde and 3% (v/v) paraformaldehyde in 0.2 M sodium cacodylate buffer (pH 7.4) for 30min, then dehydrated in graded ethanol series (50%, 70%, 80%, 90% and 100%) for 15 ​min. Dehydrated samples were dried using a critical point dryer (Leica EM CPD300). The dried samples were coated with a thin gold layer and observed with SEM (Auriga CrossBeam, Carl Zeiss microscopy GmbH, Germany).

### 2.13 Statistical analysis

The experimental data are expressed as mean ​± ​standard deviation (SD). The differences between groups in biochemical and biomechanical tests were analyzed using one-way Analysis of Variance (ANOVA) with Tukey's multiple comparison test. All p-values less than 0.05 were considered to be significant (p ​< ​0.05). Non-significant differences (ns) were indicated for p ​≥ ​0.05.

## 3 Results & discussion

### 3.1 Verification of the decellularization process

**Fig. 2:**
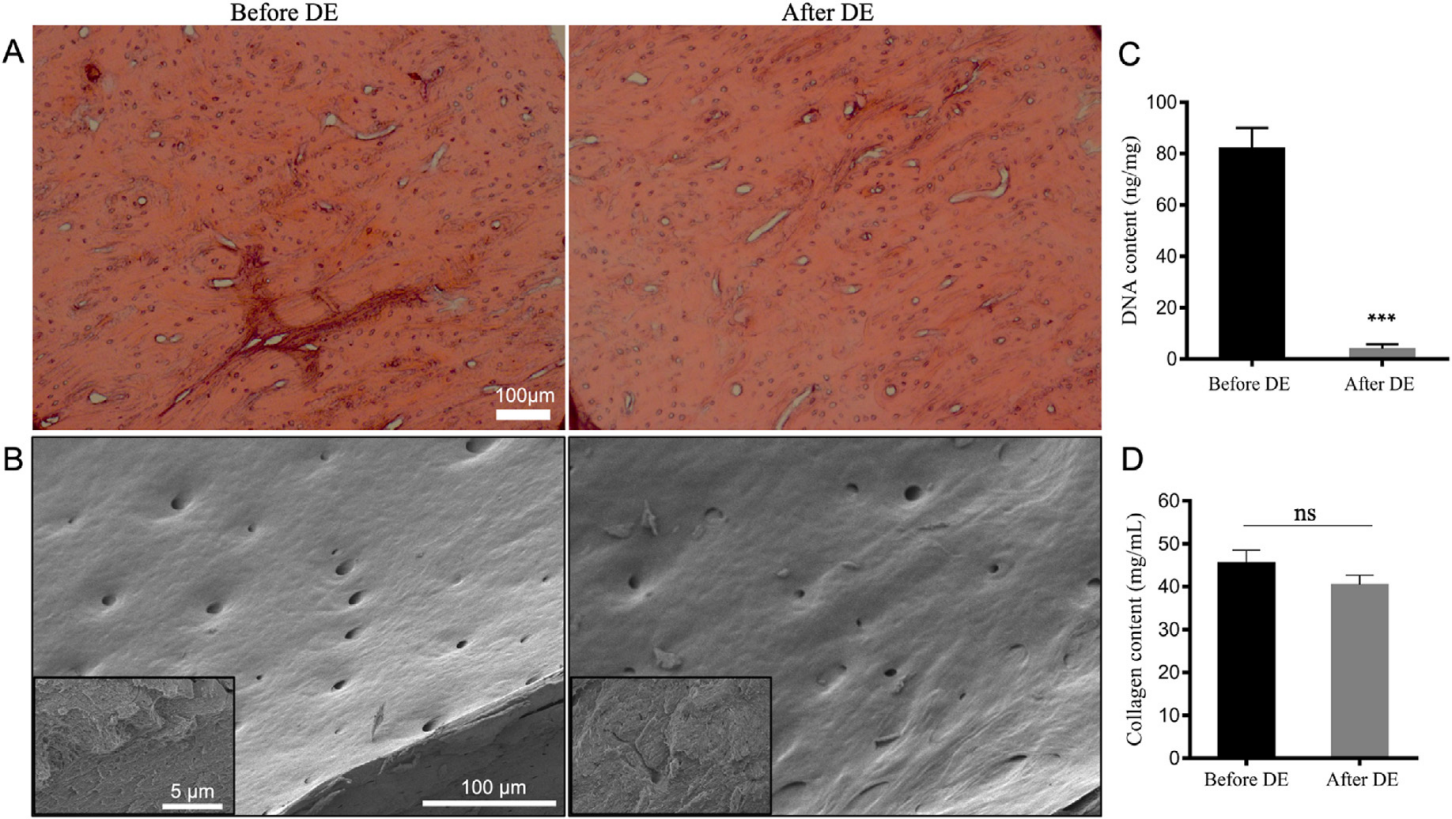
Results of the decellularization process to obtain dbPTs. (A) Histological hematoxylin & eosin staining showing cell nuclei in native bone tissue before decellularization and empty lacunae after decellularization. Scale bar: 100 ​μm. (B) SEM images of the bone tissue showing also magnified images of collagen structures. Scale bar: 100 ​μm, 5 ​μm (insets). (C) Total DNA and (D) collagen content of the bone tissues before and after decellularization. (DE: decellularization). Data are shown as mean ​± ​SD. ∗∗∗p ​< ​0.001 indicates statistically significant difference of means by one way ANOVA test.

Bone samples were evaluated histologically, morphologically, and biochemically after the decellularization process to verify the success of the decellularization protocol. Histological staining shows that round-shaped osteoblasts were homogenously distributed in untreated, native bone before decellularization, and cell lacunae were observed empty after decellularization, which indicate that cells were successfully removed from bone tissue after decellularization (Fig. 2A). SEM images indicated a typical surface morphology of the bone sample indicating also the presence of Haversian canals on the surface (Fig. 2B). In addition, collagen fibers were shown at a higher magnification, indicating that the micro histoarchitecture of the bone tissue was preserved after decellularization. Biochemical assay results confirmed the removal of DNA content. After decellularization, 95.8% reduction in DNA content was achieved. DNA content is an essential criterion for verification of the decellularization process. It was reported that the upper limit of DNA content for complete decellularization is 50 ​ng/mg [52]. The residual DNA after decellularization was much lower than the recommended upper limit. Moreover, the collagen content of bone samples did not affect the decellularization process and no statistically significant differences were found as expected. Overall, based on the conducted evaluations, it can be concluded that the decellularization process was performed successfully without any significant effect on the natural ECM structure.

### 3.2 Fabrication of GEL/dbPTs composite scaffolds

**Fig. 3:**
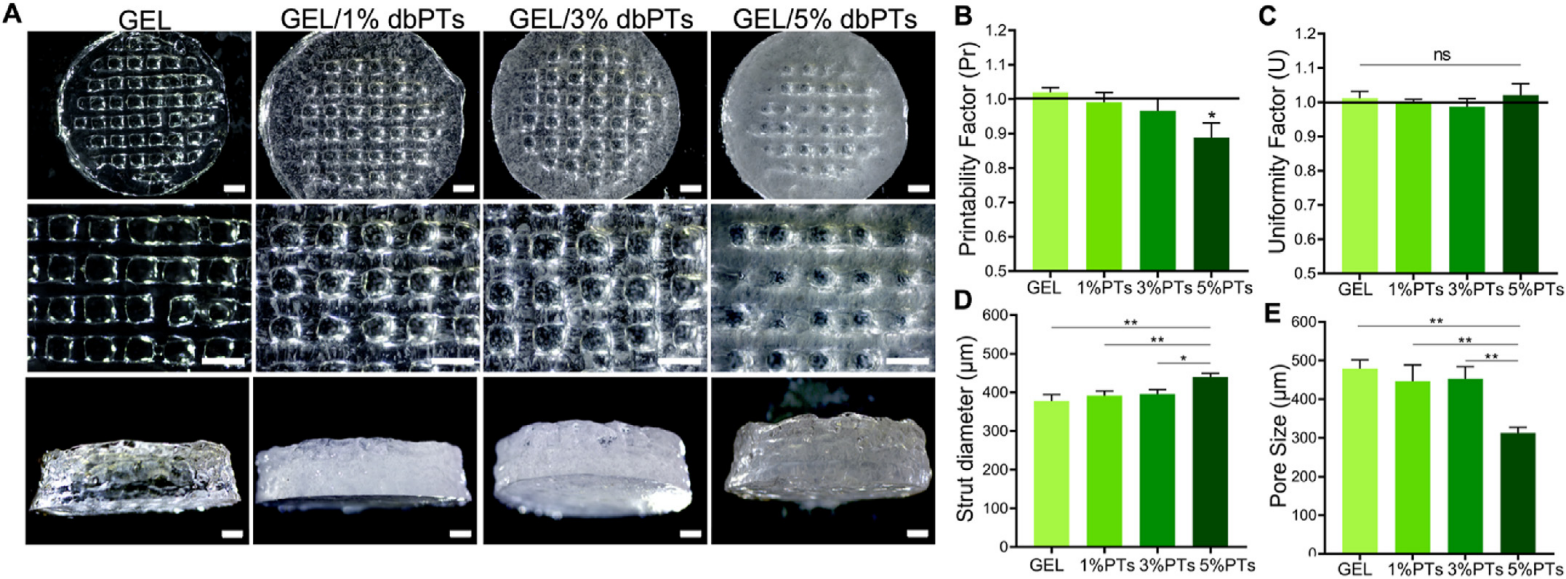
3D-printed GEL, GEL/1%dbPTs, GEL/3%dbPTs, and GEL/5%dbPTs composite scaffolds. (A) Light microscopy images of the 3D GEL/dbPTs scaffolds in top and side view after crosslinking. Scale bars: 1000 ​μm. (B–E) Printability assessments of the GEL/dbPTs scaffolds (n ​= ​4), (B) Printability factor (Pr), (C) Uniformity factor (U), (D) strut diameter, and (E) pore size of the scaffolds. Data are shown as mean ​± ​SD. ∗p ​< ​0.05 and ∗∗p ​< ​0.01 indicate statistically significant difference of means in comparison to 3D-printed GEL by one-way ANOVA tests.

This study demonstrates the fabrication of composite scaffolds composed of dbPTs and GEL by 3D bioprinting and the effect of dbPTs reinforcement on the 3D structure. GEL/dbPTs composite scaffolds containing 1%, 3%, and 5% dbPTs (wt%) were fabricated with cylindrical shape and alternating 0°/90° strut-patterns, resulting in square macropores between the strands (Fig. 3A). In the light microscopy images, dbPTs are seen in the 3D-printed structures as a white color phase. Moreover, increasing turbidity was observed with an increase in particle amount in structures. As seen in the GEL/5% dbPTs scaffold group, the scaffolds showed a white color compared to the transparent GEL scaffolds (Fig. 3A, left column), which is indicative of the higher amount of particles. The pure GEL control group showed ideal square pore geometry with uniform strut morphology, which was quantified using printability and uniformity factors (Fig. 3B and C). As described previously, the thermal pre-treatment of the GEL at 80 ​°C for 3 ​h causes partial degradation of GEL by hydrolysis, which enhances the printability characteristics [67]. This thermal modification does not change the chemical composition of the GEL, but leads to an altered molecular distribution and improvement of viscosity for 3D-printing of GEL [67,68]. With the enhancement of the printability by thermal modification of GEL, the GEL and GEL/dbPTs biomaterial inks showed high printability in the present study. However, pore morphology of GEL containing dbPTs was shown to be rounded up with increasing dbPTs concentration (Fig. 3B, E).

Printability of the biomaterial ink is an essential criterion for 3D scaffold production. Gelation of the biomaterial ink is directly related to printability. As defined by Ouyang et al. in the optimum gelation condition, the extruded filament exhibits a defined morphology with a smooth surface and constant width in three dimensions; thus, square pores and regular grids occur [61]. Conversely, when the material is in an under-gelation condition, the extruded filament exhibits a liquid-like state and the layers could fuse with each other. Thus, circular pores can occur. Pore morphology of the printed structure should exhibit a square shape, reproducing the initial G-code scaffold design, while the value of the printability factor (Pr) should be approximately one for an optimum gelation and ideal printability [61]. To assess hydrogel printability, Pr and U were calculated as a measure of pore circularity and strut homogeneity, respectively [61,62]. The quantification of Pr revealed that GEL scaffolds containing 1% and 3% dbPTs showed a similar capability to provide square pores as the pure GEL group (Pr∼1). However, the GEL/5%dbPTs showed a slight decrease in Pr with a tendency toward strut fusion, resulting in more circular pore morphology (Pr ​= ​0.9 ​± ​0.04) (Fig. 3B). As a result, the data indicate that printability is mitigated at high concentrations (≥5%), while we identified the optimal printability at particle concentrations <5% of filler content. Nevertheless, the 5% dbPT composition still exhibited a printability factor of ∼0.90 ​± ​0.04, and hence printability with sufficient shape stability for 3D printing application [61]. The uniformity of 3D-printed GEL/dbPTs scaffolds revealed that all scaffold groups have similar uniformity with no statistically significant differences (Fig. 3C). Besides, the strut diameter of printed scaffolds increased with an increasing amount of particles. The highest strut diameter was in the GEL/5%dbPTs group as 443 ​± ​3 ​μm, and statistically significant differences were found in GEL/5%dbPTs compared to other groups (Fig. 3D). The pore sizes of the 3D-printed GEL, GEL/1%dbPTs, GEL/3%dbPTs and GEL/5%dbPTs were determined as 489 ​μm, 447 ​μm, 437 ​μm and 317 ​μm, respectively, with statistically significant differences in the GEL/5%PTs group compared with the other groups (Fig. 3E). According to all printability quantification results, pristine GEL and GEL containing 1%dbPTs or 3%dbPTs scaffolds demonstrated the most uniform struts with square pore morphology, compared to the GEL/5%dbPTs. The results indicate that after a threshold of 5%dbPTs, the composite printability is mitigated. Therefore, GEL/1%dbPTs and GEL/3%dbPTs scaffolds were determined as optimal concentrations for the new biomaterial ink composition due to the well-shaped 3D structures.

**Fig. 4:**
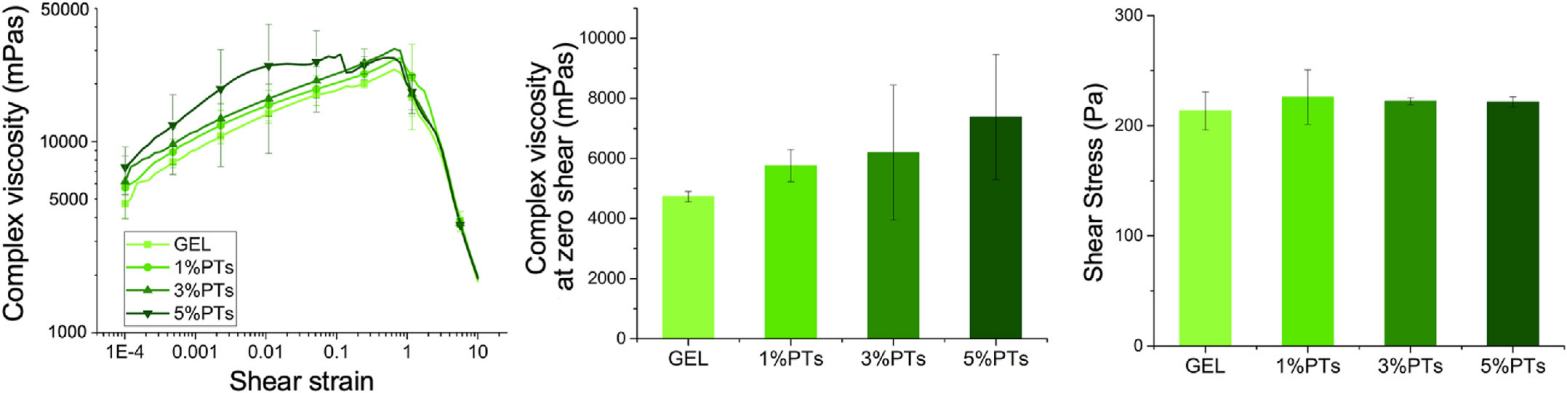
Rheological assessment of the GEL, GEL/1%dbPTs, GEL/3%dbPTs, and GEL/5%dbPTs precursors. The measurements were performed using three sample replicates (n ​= ​3), data are presented as mean ​± ​SD.

Rheological characterization of the GEL and GEL/dbPTs hydrogel precursors was performed to observe the influence of dbPT addition. The results indicate that with the addition of dbPTs, storage modulus, complex viscosity, and shear stress slightly increase (Fig. 4). Complex viscosity at rest was obtained from the average of the first measurement point (0.0001 1/s) and indicated that viscosity increased with an increasing dbPT amount in the GEL precursor. Shear stress of the samples representing the flow point of all groups was similar. dbPT containing groups showed a slight increase; however, no statistical differences were found in shear stress values. A slightly higher storage modulus was detected in the particle-incorporated hydrogel precursors with similar thixotropic behavior (Supporting information, Fig. 1).

**Fig. 5:**
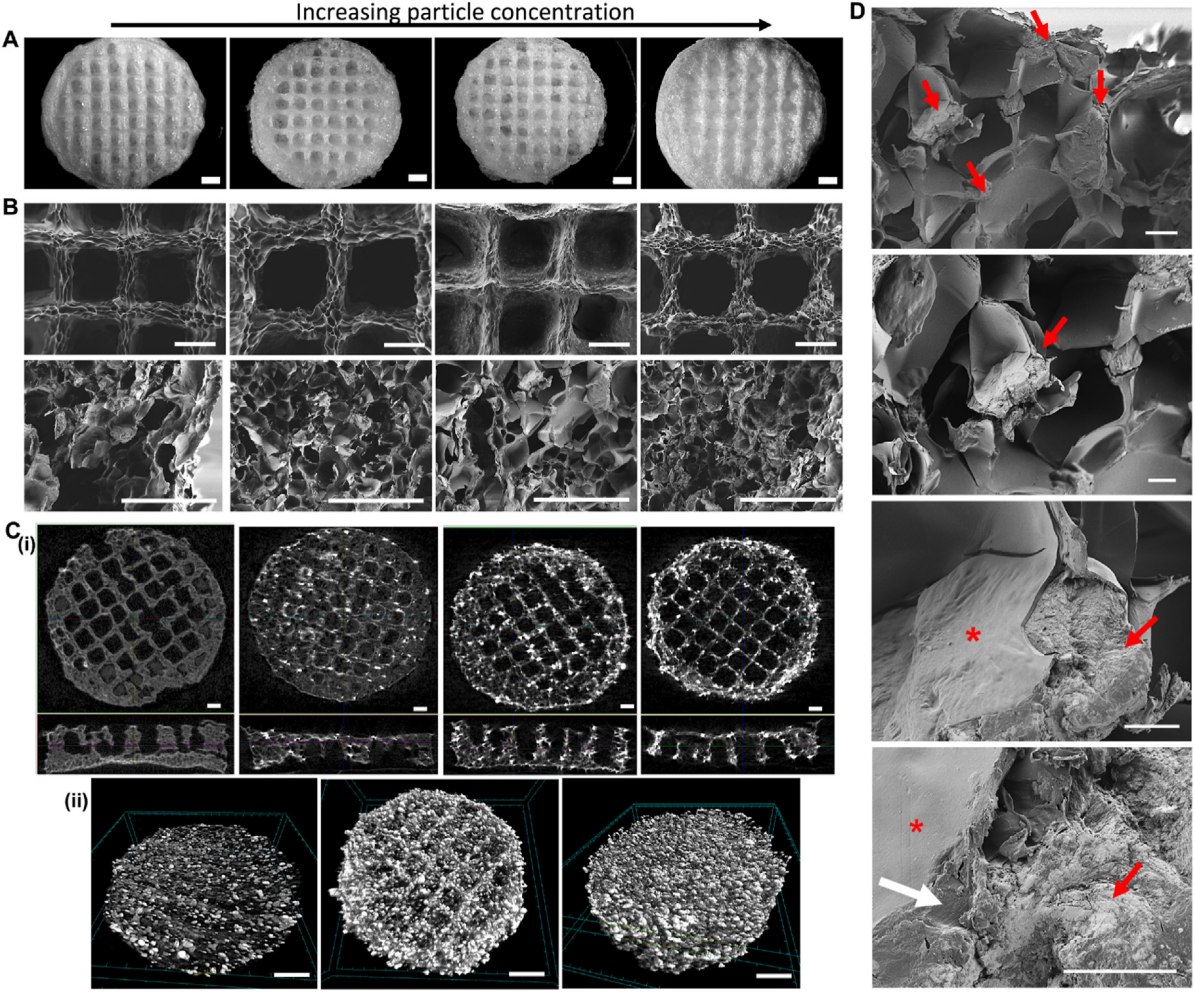
3D-printed GEL/dbPTs composite scaffolds. Scaffolds display an increasing dbPTs content in figures A, B, and C from left to right. (A) Light microscopy images of the GEL, GEL/1% dbPTs, GEL/3% dbPTs and GEL/5% dbPTs scaffolds (from left to right) after freeze-drying. Scale bars: 1000 ​μm. (B) SEM images of the GEL/dbPTs scaffolds showing the surface morphology of the scaffolds on the top view and pore structure in the cross-sectional area of the scaffolds. Scale bars: 500 ​μm. (C) μCT images of the GEL/dbPTs scaffolds with increasing dbPTs content. Images display the dbPTs distribution in the scaffolds by (i) visual analysis of cross-sectional area and (ii) volume rendering images of GEL/1%dbPTs, GEL/3%dbPTs and GEL/5%dbPTs scaffolds. Scale bars: 1000 ​μm (i) and 500 ​μm (ii). (D) Polymer-particle interaction and dbPTs bond in the GEL/1%dbPTs scaffold. Asterisk, red arrows and white arrows indicate polymer, particles and the GEL/particle interaction, respectively. Scale bars: 50 ​μm (first), 20 ​μm (second), 10 ​μm (third and fourth).

After 3D printing, GEL/dbPTs scaffolds were freeze-dried to obtain microporous scaffold structures. Fig. 5A shows light microscopy images of scaffolds in top-view. The data indicate that the pore size of the scaffolds was increased upon freeze-drying, and strut diameter was decreased due to the sublimation of the water during the freeze-drying process. After the freeze-drying process, strut diameters of GEL, GEL/1%dbPTs, GEL/3%dbPTs and GEL/5%dbPTs were determined as 259 ​μm, 284 ​μm, 322 ​μm, 430 ​μm, respectively. In addition, pore sizes of GEL, GEL/1%dbPTs, GEL/3%dbPTs and GEL/5%dbPTs were determined as 809 ​μm, 855 ​μm, 617 ​μm, 651 ​μm, respectively (Supporting information, Fig. 4). SEM images indicated open micropores on the struts as a result of the freeze-drying, while 3D-printed scaffolds have both macro (∼750 ​μm) and micropores (∼30 ​μm) after the freeze-drying procedure (Fig. 5B). The interconnected pore structure provides a large surface area and the appropriate microenvironment for cell attachment and promotes cell migration, proliferation, and transport of nutrients. Previous studies reported that pores with a diameter of ≥300 ​μm are important to allow for the connection of tissues and invasion of blood vessels into the defect area, ultimately providing vascularization, while pores around 50 ​μm diameter are advantageous to increase the surface area of scaffolds for cell attachment [[69], [70], [71]]. In our current study, it is demonstrated that the fabricated GEL/dbPTs scaffolds with micropores in a range of 20–750 ​μm are in the suitable range for bone tissue engineering applications [[69], [70], [71]]. Besides, SEM micrographs indicate that pure GEL scaffolds have a smoother pore surface in comparison to dbPTs-reinforced GEL scaffolds which have a rougher scaffold surface (Fig. 5B). Moreover, as seen in Fig. 4B, roughness of the surface was increased while increasing particle concentration. The collagen and hydroxyapatite content of the particles as well as the fibrous structure of the collagen could affect the surface morphology and lead to an increase in surface roughness.

The μCT analysis demonstrates the pore morphology of the scaffolds and the distribution of the dbPTs (Fig. 5C). dbTPs were observed as bright dots compared to the pure GEL scaffolds (Fig. 5C i), and μCT analysis confirmed the formation of an open-porous polymer network and homogeneous distribution of the particles in the whole scaffold volume (Fig. 5C ii). Slight accumulations of particles can be seen at the edges but are also associated with greater deposition of the hydrogel. The 3D analysis showed a homogeneous, Gaussian particle size distribution. The vast majority of particles in each group is around 50–100 ​μm in size (Supporting information, Fig. 2). Only a few larger particles or agglomerates could be detected. The number of detectable particles goes along with the particle concentration in the precursor. It can, therefore, be assumed that no segregation has occurred due to the printing process.

GEL is a polydisperse protein formed by irreversible acid-base hydrolysis of collagen fibrils and shows a very similar chemical composition to collagen [24]. Besides the collagen-like chemical composition of GEL, the main component of dbPTs is collagen fiber, which allow recognition and interaction in the same environment. Therefore, in order to the detailed observation of the particle-polymer interaction cross-sectional areas of the scaffolds were examined by SEM. Images demonstrated that particles were well integrated into the GEL matrix, and notably, dbPTs were observed on the pore walls on the scaffolds (Fig. 5D). This integration indicates a strong interaction between GEL and dbPTs with proper interface adhesion which could occur between organic components of the bone and GEL via strong secondary interaction [72]. Moreover, the identical chemical composition of those two materials allowed acting as a single structure while giving rise to the stabilization of the dbPTs in the GEL matrix. Furthermore, it is considered that the homogeneous distribution of the particles demonstrated in the μCT images is achieved due to the good GEL/dbPT interaction.

### 3.3 Mechanical properties of the 3D-printed scaffolds

**Fig. 6:**
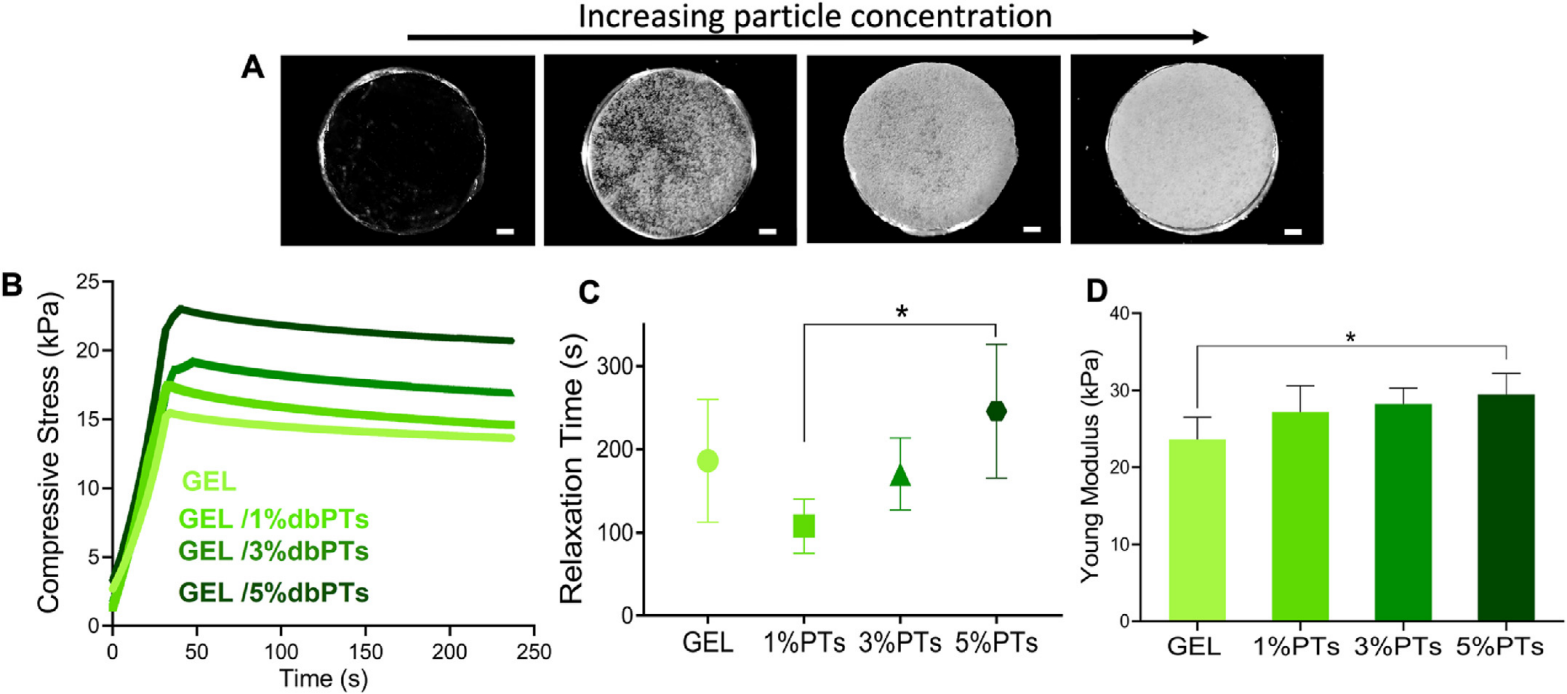
Mechanical characterization of the composite scaffolds; (A) light microscopy images of the GEL, GEL/1%dbPTs, GEL/3%dbPTs and GEL/5%dbPTs scaffolds prepared for the mechanical test (from left to right, scale bars:1 ​mm), (B) compressive stress and (C) Stress-relaxation time defined as the time after which 75% of the initial stress (upon 15% initial displacement) dissipated stress-relaxation time and (D) Young's modulus of the GEL/dbPTs scaffolds. The measurements were performed using six scaffold replicates (n ​= ​6, mean ​± ​SD). ∗p ​< ​0.05 indicates statistical differences of means in comparison to 3D-printed pristine GEL and GEL/5%dbPTs scaffolds by one-way ANOVA test.

Mechanical properties of GEL/dbPTs scaffolds were evaluated with stress-relaxation tests to determine the influence of dbPT concentration on the mechanical properties of the scaffolds. Fig. 6A depicts the light microscopy images of the composite hydrogel discs with increasing dbPT content. The compressive stress of the scaffolds over time is shown in Fig. 6B. dbPTs-reinforced scaffolds exhibited a viscoelastic behavior and stress-relaxation, because of potential matrix-reorganization over time. The stress-relaxation time was defined as the time after which 75% of the initial stress (upon 15% initial displacement) was dissipated in the scaffolds (Fig. 6C). The data indicate that GEL/1%dbPT showed the fastest relaxation in comparison to the other groups, while GEL/5%dbPT showed the slowest stress-relaxation. All groups exhibited a similar stress-relaxation time, except for a statistically significant difference between GEL/1%dbPTs and GEL/5%dbPTs. Sartuqui et al. demonstrated that the effect of hydroxyapatite crystals on mechanical properties of GEL matrix exhibited interconnected micro-and macro-porosity [73]. Particles disturbed the polymer hydro-dynamic environment and have the potential to modulate hydrogel relaxation by influencing the scaffold structure and its mechanical properties, which confirms the effect of dbPTs on scaffold relaxation [73]. The Young's modulus of the scaffolds was quantified by fitting the slope of the linear elastic deformation region from stress-strain data between 5% and 10% deformation. It was detected that the Young's modulus of the scaffolds increased with particle concentration. The highest modulus was recorded as 29 ​± ​3 ​kPa for GEL/5%dbPT scaffolds, which was significantly higher in comparison to the pure GEL control group (24 ​± ​3 ​kPa). The Young's modulus of the GEL/1%dbPT and GEL/3%dbPT scaffolds was measured as 27 ​± ​3 ​kPa and 28± 2 ​kPa, respectively (Fig. 6D). According to the mechanical test results, particle reinforcement enhanced the elastic modulus of the GEL matrix. As shown in SEM images, the proper particle-polymer interaction provided a load transfer between the two materials in accordance with the composite theory [74,75]. Thus, the mechanical properties of the GEL/dbPTs composite scaffolds were improved by this strong interface bonding. Contrarily, it has been reported that the addition of bioactive inorganic filler can result in a decrease in the mechanical properties, which may be caused by improper interface bonding between polymer and filler [76]. In our current study, dbPTs contain both inorganic and organic components due to bone nature; therefore, dbPTs appear to allow optimum bonding to a GEL matrix. Moreover, GEL has been used with different polymers or filler materials to enhance mechanical properties. Li et al. printed silk fibroin and GEL together and found that compression moduli of the scaffolds increased when the silk fibroin concentration increased; however, the water uptake and swelling ratio of the scaffolds decreased [16].

### 3.4 Physicochemical properties of the 3D-printed scaffolds

**Fig. 7:**
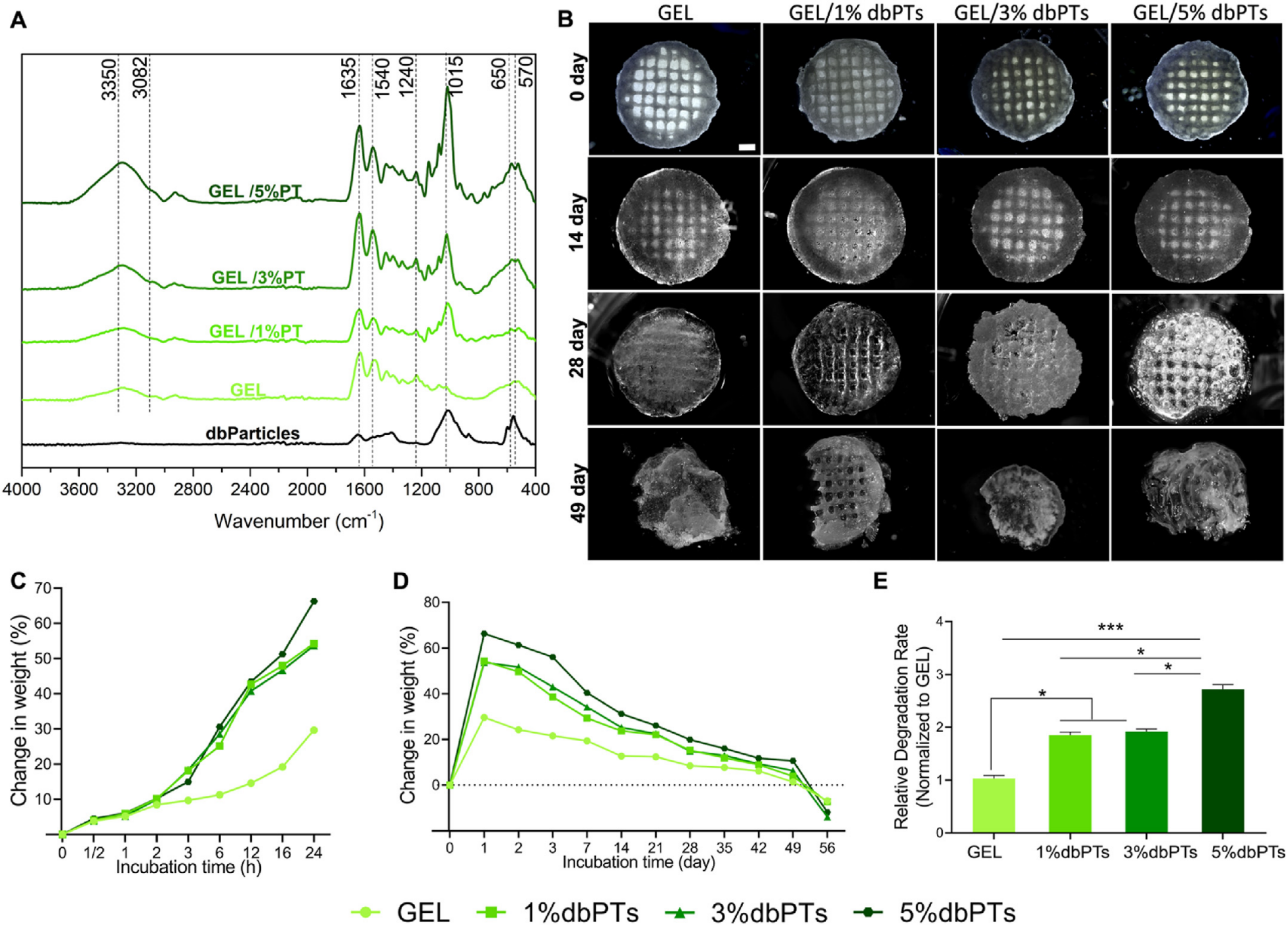
Physicochemical characterization of the 3D-printed GEL/dbPTs composite scaffolds. (A) FTIR spectrum of the dbPTs, GEL, and GEL/dbPTs scaffolds (n ​= ​3). (B) Degradation/swelling behavior of the scaffolds assessed by light microscopy images at 0, 14, 28, and 49 days of the incubation. Scale bars: 1 ​mm. Weight changes of the scaffolds (n ​= ​6, mean ​± ​SD) after (C) 24 ​h and (D) 56 days incubation period. (E) Degradation rate of the scaffolds quantified by a linear slope of the weight change between 24 ​h and 21 days with statistically significant differences (∗p ​< ​0.05, ∗∗∗p ​< ​0.001).

3D-printed GEL/dbPTs scaffolds were evaluated in terms of the assessment of the physicochemical properties by FTIR analysis and swelling/degradation study. FTIR absorbance spectra of dbPTs, GEL, GEL/1%dbPTs, GEL/3%dbPTs, GEL/5%dbPTs scaffolds showed the formation of absorbance peaks in Fig. 7A dbPTs exhibited main characteristic absorption bands of collagen, described to C=O peptide group of amide I, *N*–H bending vibration and C–N stretching vibration of amide II and C–C stretching vibrations of III of collagen which were observed at 1635 ​cm^−1^, 1540 ​cm^−1^ and 1240 ​cm^−1^, respectively. In the inorganic portion of the bone, the main detected components were phosphate and carbonate phases. The broadest absorption band at around 1015 ​cm^−1^ originated from the P–O stretching. In addition, P–O bending bands at 650 and 570 ​cm^−1^ are assigned to the O–P–O bending mode of hydroxyapatite (Ca_10_(PO4)_6_(OH)_2_) [77]. Basic characteristic peaks of GEL were seen also as amide I, II, and III, and the broad band at around 3350 ​cm^−1^ and 3082 ​cm^−1^ of GEL and GEL/dbPTs scaffolds is assigned to the stretching vibrations of N–H groups of amide A and amide B [78,79].

The swelling/degradation properties of the 3D-printed scaffolds were investigated by measuring the weight gain/loss after incubation in cell culture medium at 37 ​°C. Light microscopy images of the scaffolds on 0, 14, 28, and 49 days of the incubation are shown in Fig. 7B. All 3D-printed scaffolds were swollen for 2 days, and higher weight changes were measured on day 1 for GEL/5%dbPT scaffolds with a total weight gain of approximately 70%. GEL/1%dbPTs and GEL/3%dbPTs showed similar swelling behavior with a total weight gain of 60%, and pristine GEL scaffolds showed lower swollen behavior with a total weight gain of 30% (Fig. 7C). After the freeze-drying process, in addition to the macropores, a large surface area was obtained with the formation of the microporous structure. The large surface area provided adsorption of the liquid, which promotes the swelling of the scaffolds in the medium. It has been reported that for hydrogels left in the liquid after freeze-drying, the liquid first fills the macropores of the hydrogels and then becomes immobile by filling the micropores [80]. In GEL hydrogels, absorbed water is mainly free in the polymer network due to the highly interconnected pore structure. The interconnected pores provide a good permeability and quickly absorb the liquid from the surrounding environment [81]. Besides the microporous and interconnective pore structure, particle reinforcement influenced the swelling of the pristine GEL scaffolds. The higher swelling capacity was determined in the GEL/5%dbPTs group with a higher particle concentration (Fig. 7C). Collagen fibers present in the dbPTs also have a swelling feature as shown in previous studies [82,83]. The effects of the macro-and micro-porous structure and particle content provide an advantage and also enhance the swelling capacity of the GEL scaffolds. Although a greater swelling ratio is expected due to these advantages, it is estimated that the hydroxyapatite present in dbPTs limits the swelling capacity due to the relatively lower hydrophilicity, as reported previously. Pottathara et al. produced GEL/collagen/hydroxyapatite scaffolds by the freeze-drying method. They demonstrated that the swelling capacity of the hydroxyapatite-containing scaffolds was lower than that of GEL/collagen scaffold, which is due to reducing the water binding ability of the available surface group of collagen and GEL by the interaction/blocking of the hydroxyapatite [84].

Following the swelling, all GEL/dbPTs scaffolds started to lose mass. After 7 days, degradation progressed with a relatively constant degradation behavior and 40% mass loss was measured between 14 and 42 days of incubation (Fig. 7D). The limitation of the GEL is that the degradation process is very rapid. In the presented study, we used a higher concentration of mTG (10% w/v) to prevent rapid degradation. Therefore, 3D-printed scaffolds were stable during the long-term degradation period. Besides, our previous study demonstrated that the degradation behavior of GEL could be tailored by utilizing different mTG as a crosslinker; likewise, increasing concentration of mTG led to a stable structure with decreased degradation [85]. Both the mTG concentration and the presence of the dbPTs composed of collagen and hydroxyapatite enhanced the stability of the 3D-printed scaffolds during the long-term degradation period. After 42 days of incubation, scaffolds started to lose some parts (erosion), which is shown in Fig. 7B. Furthermore, the degradation rates of the scaffolds were determined (Fig. 7E). The higher degradation rate was measured to GEL/5%dbPTs scaffolds, showing statistical differences compared to the pristine GEL scaffolds (∗∗∗p ​< ​0.001). GEL/1%dbPTs and GEL/3%dbPTs were shown to have similar degradation rate, and a statistical difference was detected compared with pure GEL scaffolds (∗p ​< ​0.05). The swelling/degradation results indicate that the swelling of the material is enhanced upon dbPTs addition, rather than the degradation rate, as all samples start to lose their initial weight after approximately 49 days independently of particle content.

### *3.5* *In vitro* cytocompatibility assessment

**Fig. 8:**
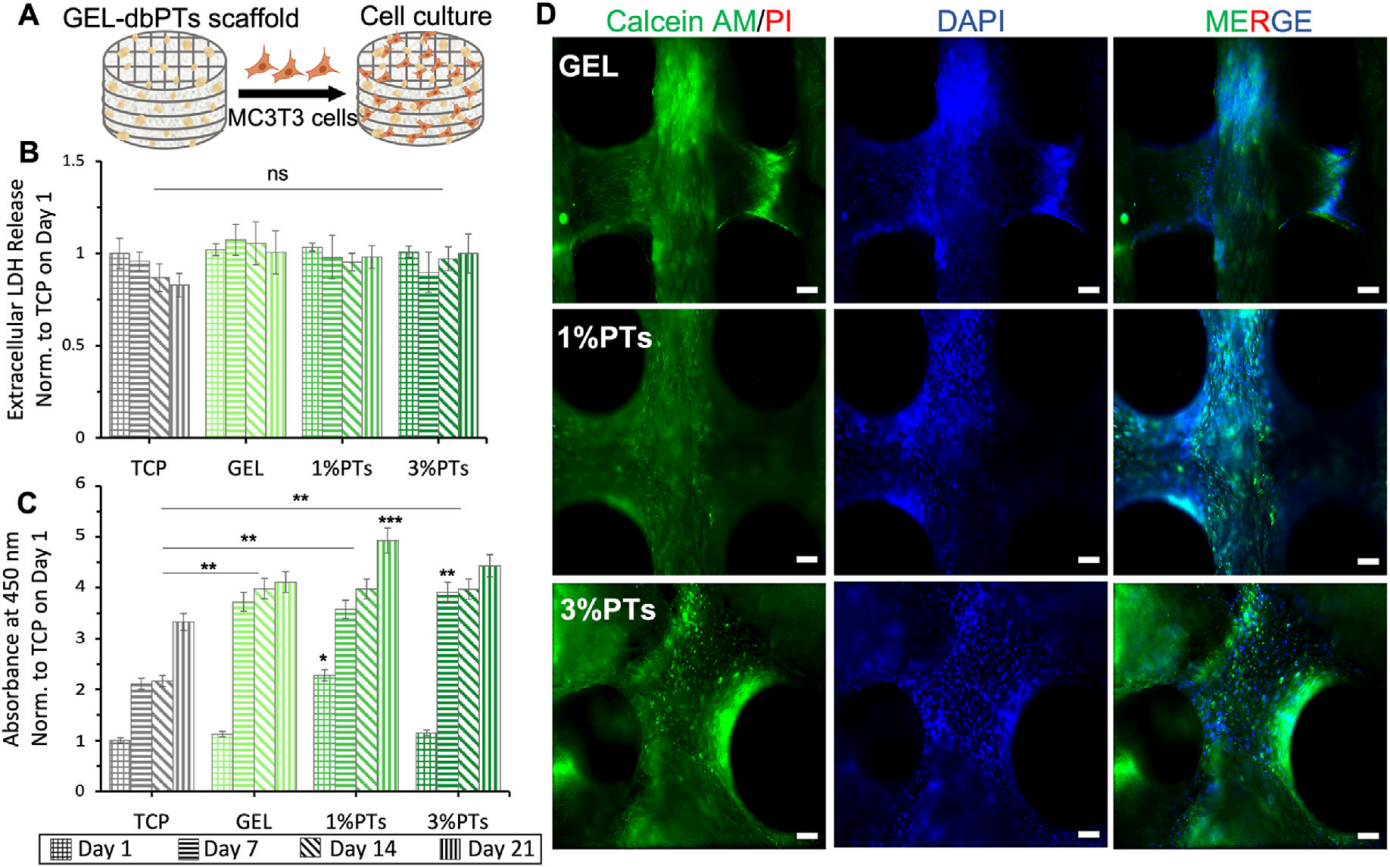
*In vitro* cytocompatibility assessment of the GEL/dbPTs composite scaffolds. (A) Schematic illustration of the cultured GEL/dbPTs scaffolds with MC3T3-E1 cells. (B) Quantification of the extracellular LDH during 21 days of incubation with no significant differences (ns) between groups on each time point (n ​= ​6). (C) Cell viability assay of MC3T3-E1 cells on the GEL/dbPTs scaffolds (n ​= ​6), normalized to TCP control on first day of incubation. Statistically significant differences were determined between groups depending on each time point. All data are represented as mean ​± ​SD. ∗∗p ​< ​0.01, ∗∗∗p ​< ​0.001 indicate statistically significant difference among means. (D) Fluorescence microscopy images of MC3T3 cells on the scaffolds cultured for 14 days. Calcein AM (green), PI (red), DAPI (blue) staining represent live, dead cells and cell nuclei respectively. (Scale bars: 100 ​μm).

*In vitro* cell culture studies were performed with MC3T3 pre-osteoblast cells in terms of evaluating the cytocompatibility and bioactivity of the GEL/dbPTs composite scaffolds (Fig. 8A). 1%dbPTs and 3%dbPTs reinforced GEL scaffolds were used for the cell culture study due to their higher printability capacity and reproducibility. Pristine GEL scaffolds and TCP were used as control groups. The cell culture assays demonstrate that MC3T3-E1 pre-osteoblast cells were grown well on all scaffold groups. To assess the potential cytotoxicity of the scaffolds, LDH release tests were performed on days 1, 7, 14 and 21 days. The results showed that released LDH levels were similar for each group, and there were no statistical differences between groups and time points which indicates that all scaffolds had no cytotoxic effect on cells (Fig. 8B). The cell viability was determined based on the metabolic activity by WST-8 assay. The results demonstrated that the metabolic activity of MC3T3-E1 pre-osteoblasts in GEL/dbPTs scaffolds was gradually increased during the culture period (Fig. 8C). The cell viability reached the highest level in all groups on day 21, and statistically significant differences were detected in all groups compared to TCP control (∗∗p ​< ​0.01). Notably, the higher viability was measured for the GEL/1%dbPTs scaffold group on day 21, and there were statistically significant differences compared to other scaffold groups (∗∗∗p ​< ​0.001).

Moreover, Live/Dead staining assay results indicated cell growth on the 3D-printed GEL and GEL/dbPTs scaffolds (Fig. 8D). MC3T3 cell spreading was favorable in all groups, and complete cell coverage was observed after 14 days of incubation. Furthermore, only live cells (green fluorescence) were presented, as it was difficult to find any dead cells (red fluorescence) even after 14 days of incubation. During the culture period, no dead cells were observed, which indicates that 3D-printed dbPTs-reinforced scaffolds are biocompatible and ideally appropriate for cell growth. According to all cytotoxicity assay results, dbPTs incorporated GEL composite scaffolds have a suitable environment for MC3T3-E1 cells and present promising features for cytocompatibility. In accordance with our results, GEL scaffolds exhibit high cellular viability and cytocompatibility for different cell types [9,86,87]. Besides the GEL cytocompatibility, cell viability was enhanced by reinforcement with dbPTs due to their inorganic hydroxyapatite crystals and collagen fibers contents. The cytocompatibility of these ingredients has been individually reported in numerous previous studies [[88], [89], [90]].

**Fig. 9:**
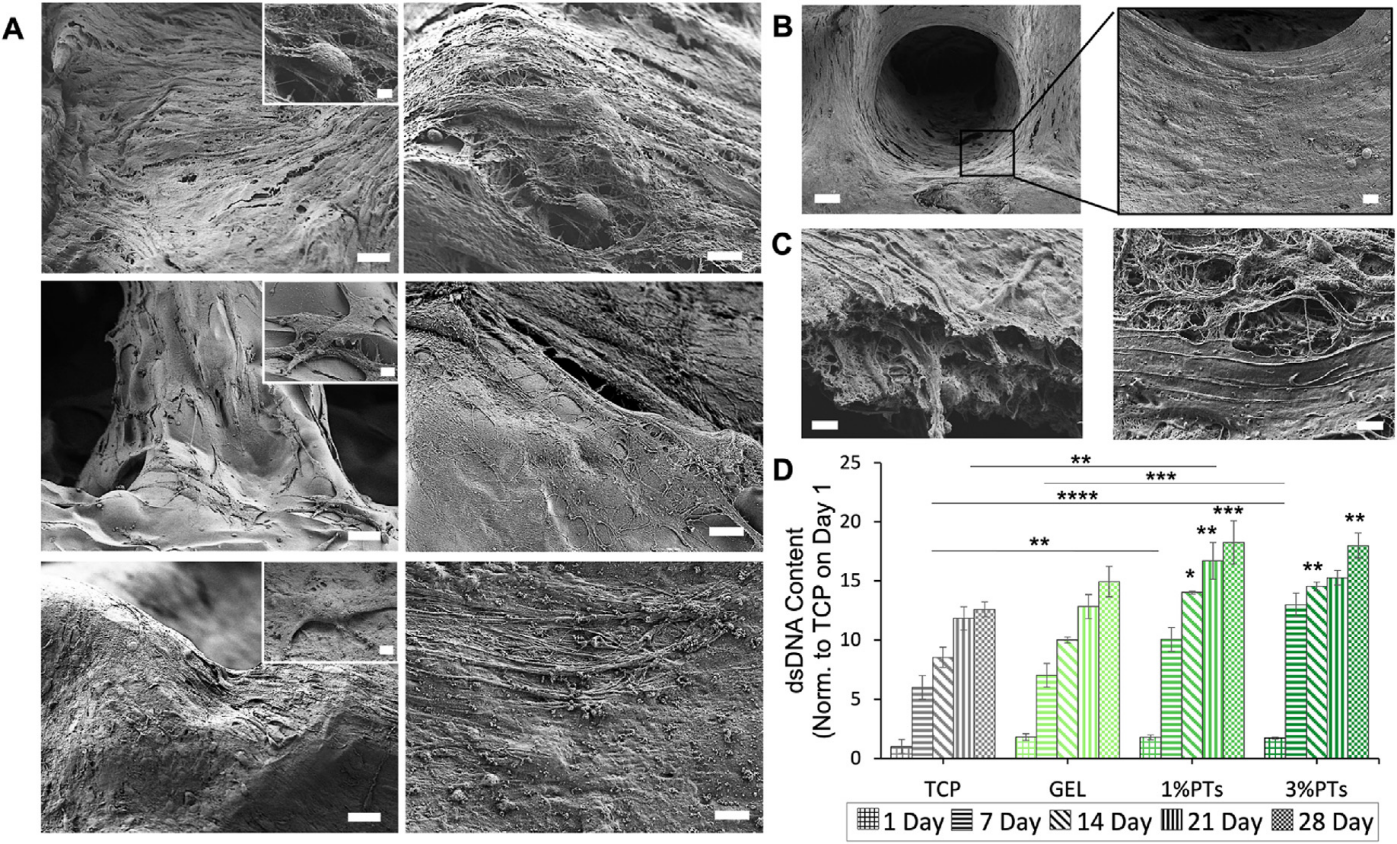
Cell proliferation and growth on the 3D-printed scaffolds. (A) SEM micrographs of MC3T3-E1 cells grown for 14 days of culture period on the GEL, GEL/1%dbPTs, and GEL/3%dbPTs composite scaffolds (from top to bottom). Cell spreading and coverage are visible for all scaffolds. Scale bars: 20 ​μm (left), 5 ​μm (right), 2 ​μm (insets). (B) Magnified SEM images of the cells which covered the pore structure of the scaffold in circular patterns. Scale bar: 100 ​μm (left), 10 ​μm (right). (C) SEM images of cells covering the surface of the scaffolds with a multilayered structure after 14 days of culture period. Scale bar: 1 ​μm. (D) Proliferation assay result of cells on the GEL/dbPTs scaffolds (n ​= ​6) quantified by PicoGreen assay normalized to TCP control on day 1. Data are shown as mean ​± ​SD and ∗, ∗∗, ∗∗∗, and ∗∗∗∗ indicate p ​< ​0.05, p ​< ​0.01, p ​< ​0.001, and p ​< ​0.0001 statistically significant data are analyzed by one-way ANOVA.

The cell attachment, proliferation and growth on the composite scaffolds were investigated by SEM analysis. The micrographs depicted spindle-shaped, elongated MC3T3-E1 cells attached, spread, and completely covering the surface of the scaffolds in all GEL and GEL/dbPTs groups (Fig. 9A). The favorable RGD peptide sequence of the GEL and cytocompatible dbPTs promoted cell adhesion. Cells were easily spread on the surface until confluency with profound cell-material interaction and even migrated into the interconnected pores. Fig. 9B shows that cells covered the pore walls and migrated into the pore during the 14 days of culture. The square pore morphology turned into a circular form which is completely covered by cells. In addition, a dense cell layer spreading on the scaffold surface with a multilayered structure and ECM production were observed by SEM imaging (Fig. 9C).

To quantify the proliferation of the cells, PicoGreen assay was performed based on the dsDNA content of the cell-seeded scaffolds during 28 days of culture period. In agreement with the cell viability test results, cells gradually proliferated on the GEL/dbPTs scaffolds (Fig. 9D). The highest proliferation of MC3T3-E1 cells was determined on GEL/1%dbPTs and GEL/3%dbPTs in comparison to the pristine GEL and TCP control at each time point. Similar to the viability test results, the highest proliferation was detected on GEL/1%dbPTs scaffolds at 21 days and 28 days. Statistically significant differences were found for both 21 days (∗∗p ​< ​0.0.1) and 28 days (∗∗∗p ​< ​0.001) compared to 1 and 7 days. Increasing cell proliferation could be directly related to the particle reinforcement and notably homogeneous distribution of the particles in the entire GEL matrix, which is reflected in the μCT images. With a similar approach, Nyber et al. showed that human adipocyte-derived stem cell proliferation and osteogenic markers such as osteonectin increased in the dbECM group compared to TCP and hydroxyapatite nonbiological additives which are widely used in bone tissue engineering applications [91].

In bone tissue engineering, numerous studies have been reported in which hydroxyapatite, bioglass, or silicate-like inorganics (as bioactive additives), and also collagen (as a supportive biopolymer) are incorporated into polymer matrices in order to promote osteogenic activity and to mimic bone tissue [[84], [92], [93], [94], [95], [96]]. In addition to the successful results of those studies, our study here presents decellularized bone particles composed of both hydroxyapatite and collagen as a natural additive. The incorporation of dbPTs into the GEL matrix enhanced cell adhesion, proliferation, and migration on/into the 3D composite scaffolds. Similar to our results, Hung et al. used dbECM to produce hybrid PCL-dbECM scaffolds by 3D printing technique and demonstrated better cell adhesion on the dbECM containing scaffolds compared to control PCL [97]. This enhancement in cellular behavior comprising cell attachment, proliferation, and maintained viability, was attributed to the presence of collagen fibers and natural hydroxyapatite crystals in dbECM particles. It should be highlighted that the main focus of the study was the development and characterization of a 3D-printable biomaterial ink with potential for bone engineering. While the data demonstrate cytocompatible materials with suitable cell-material interaction (of pre-osteoblast MC3T3-E1 cells), the potential of the materials for osteogenic differentiation was not in the scope of the presented study and requires future analyses.

**Fig. 10:**
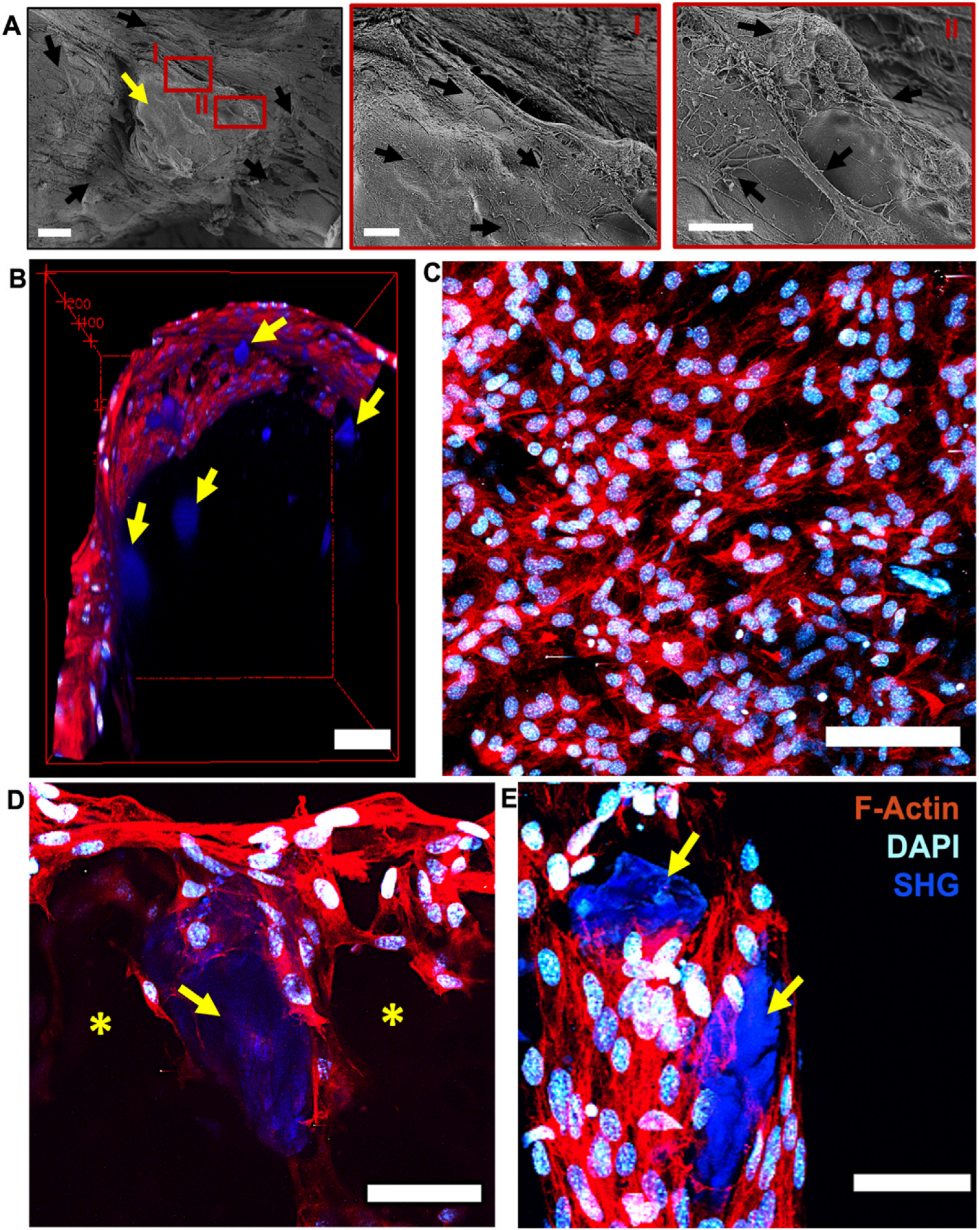
Cell and particle interaction on the GEL/dbPTs composite scaffolds at 14 days of incubation period. (A) SEM images of the MC3T3-E1 cells (black arrows) that interacted with particles (yellow arrow) on GEL/dbPTs scaffolds. Scale bar: 20 ​μm (left), 5 μm (right). (B–E) Multiphoton microscopy images, cyan ​= ​DAPI, red ​= ​F-Actin, blue ​= ​SHG. (B) 3D view of cells growing on GEL/dbPTs-composite scaffolds (yellow arrows indicate particles in blue). Scale bar: 100 ​μm (scale bar indicated by red cage) (C) Top of scaffolds covered with cell layer. Maximum intensity projection. Scale bar: 100 ​μm. (D) 3D view of cells growing on porous scaffolds and around dbPTs in blue (yellow stars and arrows indicate pore and particle). Scale bar: 50 ​μm ​(E) Cells interacting with decellularized bone particles (yellow arrows). Maximum intensity projection. Scale bar: 50 ​μm.

To obtain further insight into the cell-material interaction, cell-seeded scaffolds were observed by SEM and multiphoton microscopy. The combination of SEM analysis and multiphoton microscopy results demonstrated effective cell-particle and cell-material interactions. SEM images displayed that the cells covering the scaffold surface surrounded the particles and spread on the particles as well. Attached and elongated MC3T3-E1 cells were observed on the particle surface (Fig. 10A). Similar attachment behavior was indicated using multiphoton microscopy which demonstrated that cells surrounded and attached to the particles in the GEL/dbPTs scaffold (Fig. 10 B, D, E). DAPI staining showed the nucleus of the cells as cyan, and F-actin staining indicated the cytoskeleton of the cells as red. dbPTs showed a bright second harmonic generation signal (SHG), which may be a result from fibrous collagen in the dbPTs (Fig. 10 B, D, E blue). Multiphoton imaging could confirm that cells grew inside the porosity of the hydrogels which was created by the freeze-drying process (Supporting information Video 3, 4). The data indicate cells covering the scaffold surface as well as cell migration into pore structures of the scaffolds, interacting with the particles (Fig. 10 B, C, D, E). This cell adhesion behavior indicated that cells interact with dbPTs in the GEL matrix, which may be a result from the fibrous collagen content of the particles which facilitates cellular attachment on the particles. The results confirm that the GEL/dbPT composite facilitates cell adhesion and cell-material interaction by both, cell-adhesive enzymatically cross-linked GEL and dbPT particles. Since the particles were homogeneously distributed in the GEL matrix, as shown in μCT images, cells were homogeneously distributed on the scaffolds surface and also interacted with the dbPTs in the 3D structure. While the data indicate that dbPT concentrations >5% mitigate printability, in vitro experiments highlighted that dbPT concentrations <5%, with high biomaterial ink printability, showed no cytotoxic effects (Fig. 8 B, D) by the particles but allowed for cell proliferation (Fig. 9D) and effective cell interaction with the dbPTs (Fig. 10). During scaffold degradation, porosity in the scaffold may increase that can lead to higher exposure to scaffold surface area and particles inside the biomaterial, which then can lead to homogenous cell-material adhesion over hydrogel degradation over time. Yung et al. investigated the behavior of cells encapsulated in GEL hydrogels crosslinked by mTG and demonstrated that cells could quickly move through the GEL during degradation [98]. Yang et al. also showed the proliferation and migration of adipocyte-derived stem cells in GEL/mTG hydrogels as a cell vehicle biomaterial for bone regeneration [99]. Consistent with the literature, our study exhibited cell-matrix and cell-dbPTs interaction in 3D-printed composite scaffolds. dbECM has excellent potential for bone tissue engineering and regenerative medicine. dbECM can contribute to cell proliferation and osteogenic differentiation due to its inorganic hydroxyapatite and organic matrix composition [100]. Hung et al. showed that 3D-printed PCL-dbECM scaffolds had improved cell response in comparison to pure PCL [97]. Moreover, it was indicated that higher cell adhesion and better biocompatibility features of dbECM compared with tricalcium phosphate and hydroxyapatite in 3D-printed PCL scaffolds [91]. We demonstrated significant interaction of cells on 3D-printed GEL/dbPTs composite scaffolds with homogenous particle distribution. Preserving the natural collagen and hydroxyapatite content of the dbPTs after decellularization using 0.1% SDS, which is the lowest concentration according to literature, improved the cell attachment, proliferation, migration as well as mechanical and physicochemical properties.

Supplementary video related to this article can be found at https://doi.org/10.1016/j.mtbio.2022.100309

The following is/are the supplementary data related to this article:

Video 3 3 Video 3

Video 4 4 Video 4

In this study, the effect of dbPTs in GEL hydrogel was evaluated in terms of printability, mechanical, and physiochemical properties as well as cytocompatibility. We showed that 3D-printed GEL/dbPTs composite scaffolds with high macro/micro porosity provide a favorable environment for MC3T3-E1 cells. Preserving the natural collagen and hydroxyapatite content of the dbPTs after decellularization using 0.1% SDS, which is the lowest concentration according to literature [56,57,97,101], improved the biological activity as well as the mechanical and physicochemical properties of the scaffolds. We present a minimalistic hydrogel formulation composed of GEL (already available as FDA approved composition) [26,27], dbPTs, and a crosslinking approach based on mTG (already available with FDA approval) [60], which is envisioned to have potential for clinical translatability in comparison to complex hydrogel systems involving components that are not available as clinical grade materials. As a result, the composite GEL/dbPTs scaffolds introduced in this study may have potential for bone engineering. Future work should include the detailed investigation of the cellular response based on gene expression and bioactivity studies to assess the effect of dbPTs on osteogenesis that represent important hallmarks for a success of the here presented materials for bone tissue engineering. In addition, smaller bone particles maybe considered to improve printability enabling higher concentrations of particles in the biomaterial ink.

## 4 Conclusions

The study demonstrated the successful fabrication of composite scaffolds composed of GEL and dbPTs by 3D printing technology. dbPTs were homogeneously distributed in GEL hydrogels and exhibited significant interaction with the polymer matrix. Fabricated 3D-printed GEL/dbPTs composite scaffolds displayed high porosity with macro and micro porous structure, providing a favorable environment for MC3T3-E1 cells. Besides the nontoxicity of the composite scaffolds, particle incorporation increased cell attachment, proliferation and migration. Complete coverage of the scaffold surface by cells was displayed. The approach of using dbPTs as a natural collagen and hydroxyapatite source in 3D printing techniques using GEL matrix provides great interaction with cells, which suggests the potential application of the scaffolds in bone tissue engineering.

## Author contributions

A.K.: conceptualization, methodology, investigation, material and experimental design, experiments, data analysis, writing, review, and editing. T.D.: conceptualization, methodology, material and experimental design, SEM analysis, Multiphoton microscopy, Multiphoton microscopy data processing, review and editing. C.P. and H·S.: rheology and μCT analysis, review and editing. D.S.: Multiphoton microscopy. O.F.: Multiphoton microscopy, review and editing. F.T.: conceptualization, methodology, review and editing, supervision. A.R.B.: conceptualization, methodology, resources, review and editing, supervision.

## Data availability statement

Data supporting the reported results can be provided by the corresponding author upon request.

## Declaration of competing interest

The authors declare that they have no known competing financial interests or personal relationships that could have appeared to influence the work reported in this paper.
