# Supplementary material for: 3D printed gelatin/decellularized bone composite scaffolds for bone tissue engineering: Fabrication, characterization and cytocompatibility study
Source: Mater Today Bio. 2022 Jun 6;15:100309. doi: 10.1016/j.mtbio.2022.100309 (PMC9213825; doi:10.1016/j.mtbio.2022.100309)
Supplement: Multimedia component 1 [file mmc1.docx]

**[Supporting Information]**

**3D Printed Gelatin/Decellularized Bone Composite Scaffolds for Bone Tissue Engineering: Fabrication, Characterization and Cytocompatibility Study**

Aylin Kara^a,b^, Thomas Distler^b^, Christian Polley^c^, Dominik Schneidereit^d^, Hermann Seitz^c^, Oliver Friedrich^d^, Funda Tihminlioglu^e^, Aldo R. Boccaccini^b*^

^a^İzmir Institute of Technology, Department of Bioengineering, İzmir, 35433, Turkey

^b^Institute of Biomaterials, Department of Material Science and Engineering, Friedrich-Alexander-University Erlangen-Nuremberg, Erlangen 91058, Germany

^c^Chair of Microfluidics, Department of Mechanical Engineering, University of Rostock, Rostock 18059 Germany

^d^Institute of Medical Biotechnology, Department of Chemical and Biological Engineering, Friedrich-Alexander-University Erlangen-Nuremberg, Erlangen 91052 Germany

^e^İzmir Institute of Technology, Department of Chemical Engineering, İzmir 35433 Turkey,

Table 1. Printing parameters of GEL and dbPTs incorporated GEL

|  | Extrusion pressure  (kPa) | Nozzle velocity  (mm/s) | Temperature  (°C) |
| --- | --- | --- | --- |
| GEL | 120 | 5 | 25 |
| GEL/1%dbPTs | 135 | 5 | 25 |
| GEL/3%dbPTS | 160 | 5 | 25 |
| GEL/5%dbPTS | 180 | 5 | 25 |


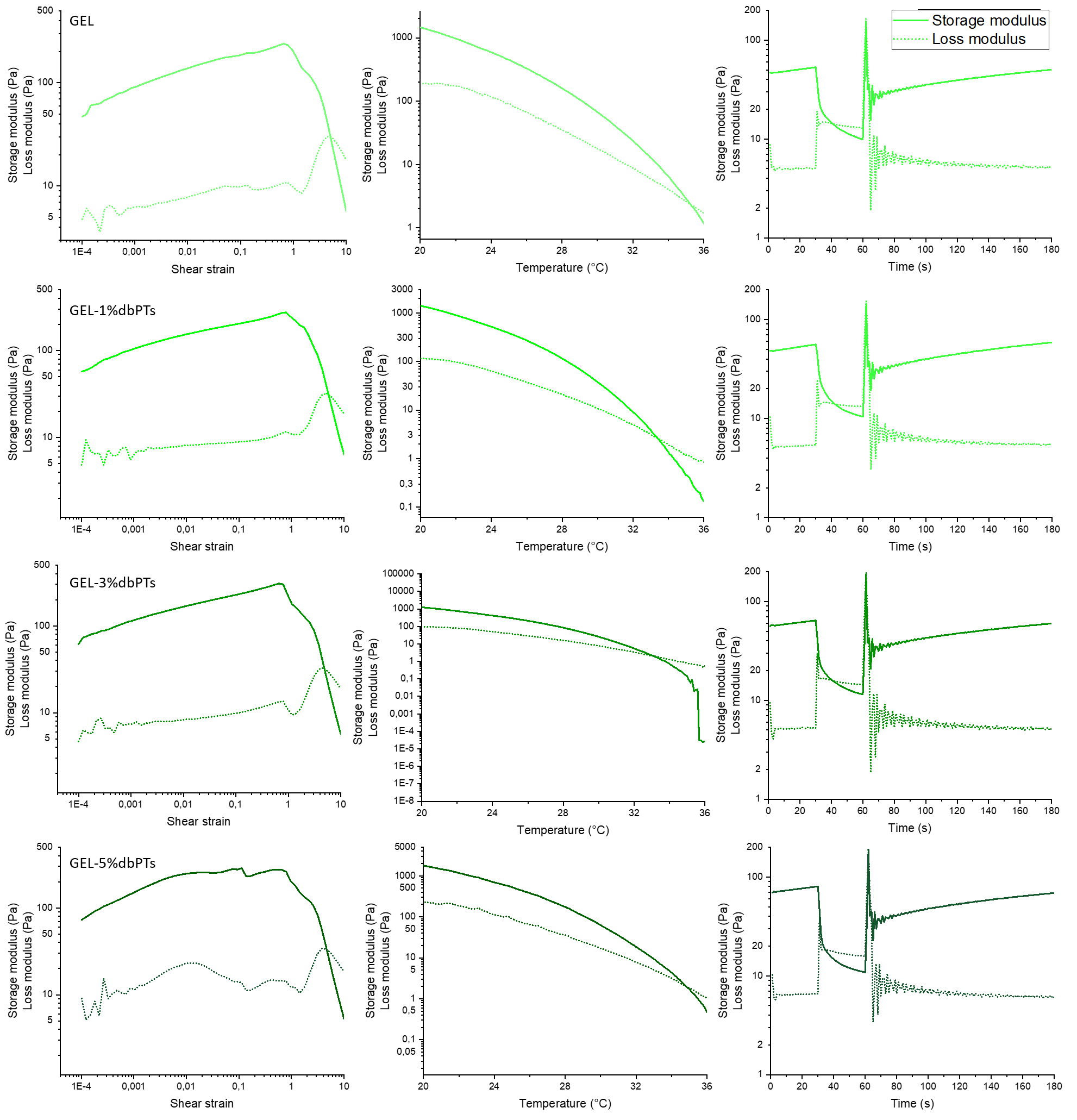


**Figure SI 1.** Rheological test results of the GEL and dbPTs incorporated hydrogel precursors. The measurements were performed using three sample replicates (n=3), data are presented as mean.


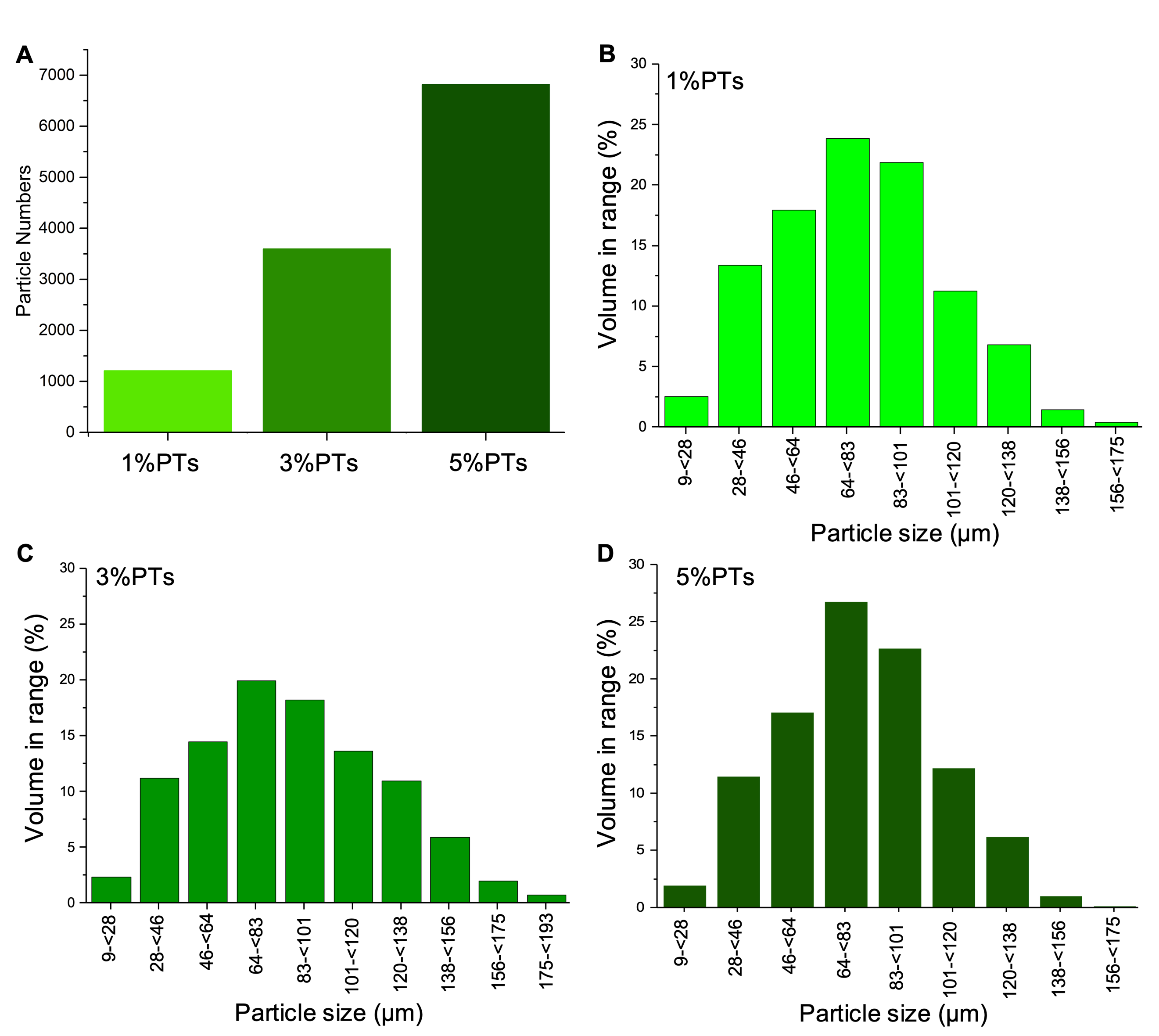


**Figure SI 2.** Evaluation of the particle distribution obtained from µCT data. A) Particle numbers in the GEL-1%dbPTs, GEL-3%dbPTs, and GEL-5%dbPTs scaffolds. Volume in range (%) analysis for (B) GEL-1%dbPTs, (C) GEL-3%dbPTs, and (D) GEL-5%dbPTs scaffolds.

**
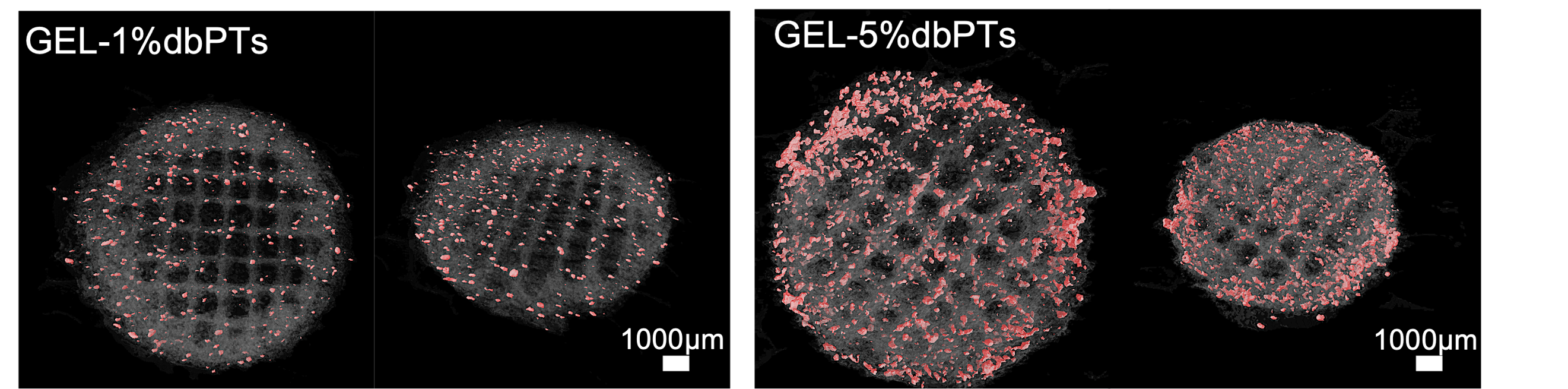
**

**Figure SI 3.** Surface render analysis of the particles (red) in the 3D printed scaffolds.

**
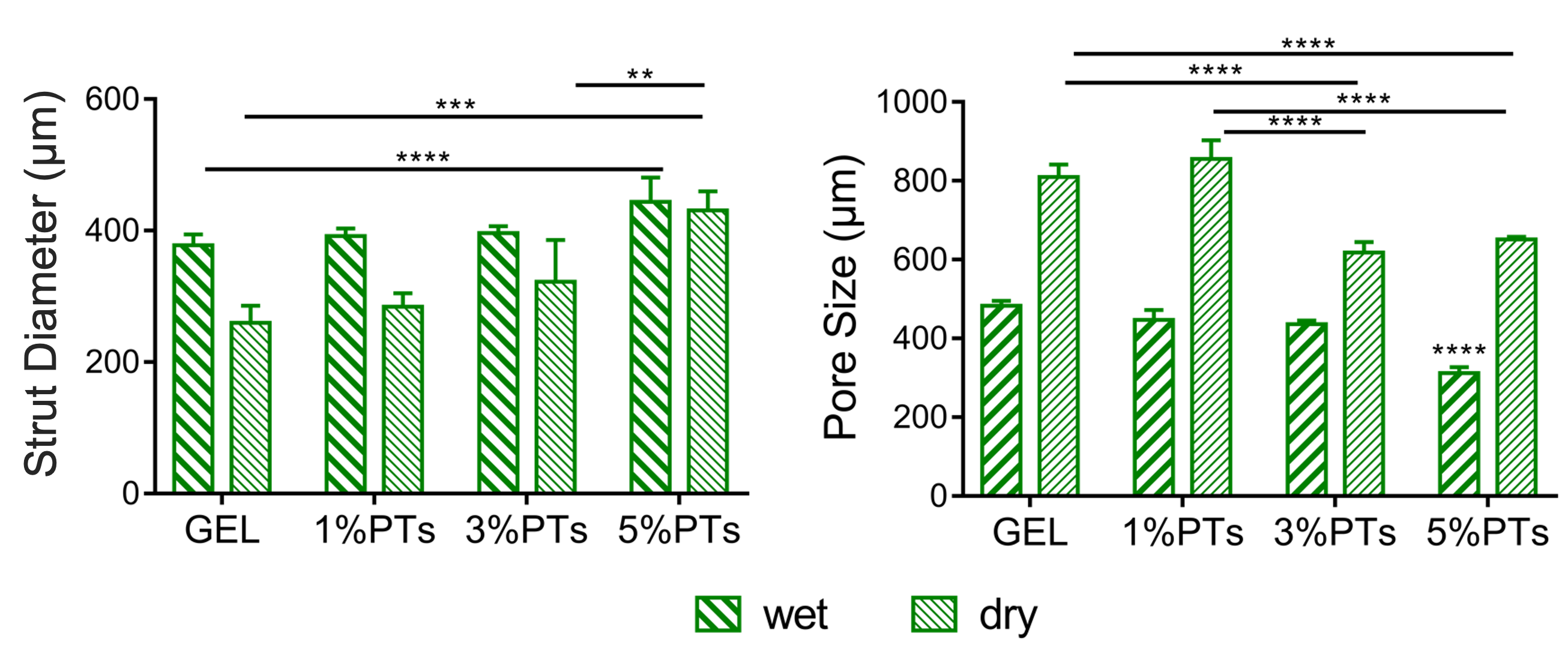
**

**Figure SI 4.** Strand diameter and pore size quantification of the 3D-printed GEL, GEL-1%dbPTs, GEL-3%dbPTs, and GEL-5%dbPTs scaffolds before (wet) and after (dry) freeze-drying process. The measurements were performed using four sample replicates (n=4). Data are presented as mean±SD. **p<0.01, ***p<0.001 and ****p<0.0001 indicate statistically significant difference of means in comparison all groups by one-way ANOVA tests.

**
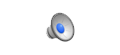
**

**Video SI 1.** μCT video of 3D printed GEL-1%dbPTs scaffold showing the porosity and distribution of the particles

**
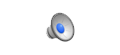
**

**Video SI 2.** μCT video of 3D printed GEL-5%dbPTs scaffold showing the porosity and distribution of the particles

**
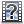
**

**Video SI 3.** Multiphoton microscopy video of GEL scaffolds at 14 days of incubation period. 3D view of cells grew inside the scaffold pores. White=DAPI (nuclei), red=rhodamine phalloidin (F-Actin), blue= autofluorescence (scaffold), black=pores. Scale bar = 100µm.

**
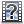
**

**Video SI 4.** Multiphoton microscopy video of GEL scaffolds at 14 days of incubation period. 3D view of cells grew inside the scaffold pores. White=DAPI (nuclei), red=rhodamine phalloidin (F-Actin), blue= autofluorescence (scaffold), black=pores. Scale bar = 100µm.

**
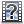
**

**Video SI 5.** Multiphoton microscopy video of GEL-1%dbPTs scaffolds at 14 days of incubation period. 3D view of cells growing on composite scaffolds with particle interaction. White=DAPI (nuclei), red=rhodamine phalloidin (F-Actin), blue= SHG (dbPTs). Scale bar = 50µm.

**
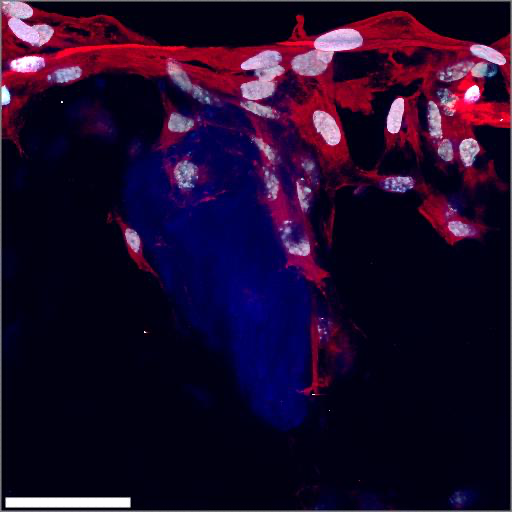
**

**Video SI 6.** Multiphoton microscopy video of GEL-3%dbPTs scaffolds at 14 days of incubation period. 3D view of cells growing on composite scaffolds with particle interaction. White=DAPI (nuclei), red=rhodamine phalloidin (F-Actin), blue= SHG (dbPTs). Scale bar = 100µm.
